# High-precision chemical quantum sensing in flowing monodisperse microdroplets

**DOI:** 10.1126/sciadv.adp4033

**Published:** 2024-12-11

**Authors:** Adrisha Sarkar, Zachary R. Jones, Madhur Parashar, Emanuel Druga, Amala Akkiraju, Sophie Conti, Pranav Krishnamoorthi, Srisai Nachuri, Parker Aman, Mohammad Hashemi, Nicholas Nunn, Marco D. Torelli, Benjamin Gilbert, Kevin R. Wilson, Olga A. Shenderova, Deepti Tanjore, Ashok Ajoy

**Affiliations:** ^1^ Department of Chemistry, University of California, Berkeley, Berkeley, CA 94720, USA.; ^2^ Chemical Sciences Division, Lawrence Berkeley National Laboratory, Berkeley, CA 94720, USA.; ^3^ Advanced Biofuels and Bioproducts Process Development Unit (ABPDU), Biological Systems and Engineering Division, Lawrence Berkeley National Laboratory, Berkeley,CA 94720, USA.; ^4^ Adamas Nanotechnologies Inc., Raleigh, NC 27617, USA.; ^5^ Energy Geoscience Division, Lawrence Berkeley National Laboratory, Berkeley, CA 94720, USA.; ^6^ CIFAR Azrieli Global Scholars Program, 661 University Ave, Toronto, ON M5G 1M1, Canada.

## Abstract

A method is presented for high-precision chemical detection that integrates quantum sensing with droplet microfluidics. Using nanodiamonds (ND) with fluorescent nitrogen-vacancy (NV) centers as quantum sensors, rapidly flowing microdroplets containing analyte molecules are analyzed. A noise-suppressed mode of optically detected magnetic resonance is enabled by pairing controllable flow with microwave control of NV electronic spins, to detect analyte-induced signals of a few hundredths of a percent of the ND fluorescence. Using this method, paramagnetic ions in droplets are detected with low limit-of-detection using small analyte volumes, with exceptional measurement stability over >10^3^ s. In addition, these droplets are used as microconfinement chambers by co-encapsulating ND quantum sensors with various analytes such as single cells, suggesting wide-ranging applications including single-cell metabolomics and real-time intracellular measurements from bioreactors. Important advances are enabled by this work, including portable chemical testing devices, amplification-free chemical assays, and chemical imaging tools for probing reactions within microenvironments.

## Teaser

Droplet microfluidics paired with diamond quantum sensing enables sensitive analyte detection for chemical and biological systems.

## INTRODUCTION

Quantum sensing (*1*) is rapidly reshaping our ability to discern chemical processes with high sensitivity and spatial resolution, with the potential to impact a range of disciplines from synthesis to bioengineering (*2*). Specifically, sensors based on nitrogen-vacancy (NV) defects in diamond (*3*, *4*) translate optically addressable electronic spin state information into detectable fluorescence signals in a manner that is sensitive to the local chemical environment. This has led to diverse applications, including in-cell thermometry (*5*–*8*) and reactive oxygen species (ROS) detection (*9*, *10*), high-sensitivity lateral flow assays (*11*), and nuclear magnetic resonance (NMR) measurements in picoliter volumes (*12*, *13*), marking an exciting leap in precision measurement technologies.

Traditionally, quantum sensing for chemical analysis has relied on single crystals hosting shallow NV centers (*14*, *15*). High-throughput analysis faces challenges due to the small (millimeter-scale) sizes of these crystals, their substantial cost, and the need for precise crystal orientation (*16*). Only a fraction of the diamond crystal is used for sensing, and analyte molecules are required to flow over its surface, necessitating complex integration of microfluidic structures directly onto the diamond (*17*–*20*).

Nanodiamond (ND)–based sensing offers a compelling alternative as they are inherently deployable and can yield spatially selective sensing in or near targeted volumes of interest. NDs are also low cost, nonreactive, and bio-inert (*21*, *22*), and for <40-nm particles, a substantial proportion of their NV centers can interact with external analytes. Advances in ND chemistry have facilitated surface functionalization to control surface charge, hydrophilicity, or hydrophobicity and for targeting to proteins or cellular organelles (*23*–*28*).

Despite these advantages, ND-based sensing is fraught with challenges. Heterogeneity in particle size (*29*), shape, and NV center coherence times leads to large statistical errors in multiparticle analysis. In addition, fluorescence fluctuations arise from different particle orientations and spatial variations in the materials in which the particles are targeted (*30*, *31*). Overcoming these challenges is essential to achieving high-precision quantum sensing.

Here, we directly address these challenges by deploying NDs within flowing, monodisperse, picoliter-volume microdroplets (*32*, *33*) that host analyte molecules. Rapid movement of the ND particles within the droplets effectively averages out their heterogeneity and ensures close interaction with the analytes. We take advantage of stable and controllable flow afforded by droplet microfluidics to develop a method for background-free quantum sensing at high-throughput. In addition, the dynamic nature of the flowing droplets yields exceptional measurement stability, resistant to experimental variations and temperature shifts. We demonstrate this stability over >10^3^ s measurement and across ∼10^5^ droplets, greatly surpassing the typical stability in conventional quantum sensing experiments (*34*, *35*). In addition, the ND sensor volumes required are minuscule, amounting to 25 μl for an hour of analysis over hundreds of thousands of droplets.

Our work, therefore, introduces a platform technology fusing quantum sensing and droplet microfluidics, and is marked by several novel aspects. Picoliter-scale droplets can function as microscopic confinement chambers, encapsulating diverse analytes, ranging from single cells to chemical reaction products, and can stably accommodate a broader range of concentrations than bulk solutions (*36*). The droplets are precisely controllable in terms of diameter, charge, and environmental conditions, and their movement under flow enhances sensor-analyte mixing (*37*, *38*). This approach is also amenable to digital control techniques for droplet “arithmetic,” including mixing, collisions, and sorting, further enhancing their application in quantum sensing.

## RESULTS

### ND loading in microdroplets

**Fig. 1.**
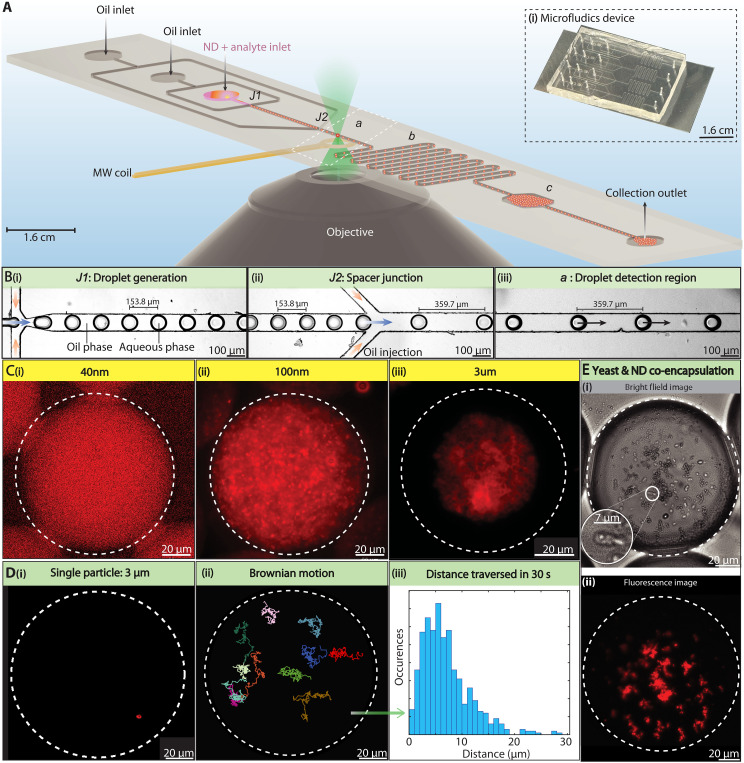
NDs in droplets. (**A**) Schematic of microfluidic chip comprising two inlets for oil and one for water (gray circles). Analyte of interest and NDs are mixed into the latter. Chip features two focusing junctions, $J_{1}$ and $J_{2}$. $J_{1}$ generates ND-filled water droplets, while $J_{2}$ regulates droplet spacing. Droplets are analyzed in a region (dashed region a) over an objective lens and MW coil, followed by a circuitous region to induce additional mixing within droplets (region b) and storage in a collection chamber (region c). (i) Inset: Photograph of chip; water and oil are delivered via narrow capillaries. See the Supplementary Materials for fabrication details. (**B**) Bright-field images with detailed views of the device regions. (i) Droplet generation at $J_{1}$ occurs by pinching water flow by oil (blue and orange arrows respectively) through an orifice. (ii) Spacer junction $J_{2}$ allows adjustment of droplet spacing via oil-flow (arrows). Here, interdroplet distance is changed ≈154 → 360 μm. (iii) Analysis region a: Droplets maintain a consistent velocity and separation downstream and are analyzed in flow. (**C**) Fluorescence images of droplets containing NDs of various sizes, (i) 40-nm-, (ii) 100-nm-, and (iii) 3-μm-diameter particles. Dashed lines outline the droplet for clarity. (**D**) Tracking single NDs in droplets. (i) Single 3-μm particle encapsulated within a droplet. (ii) Tracked motion of 100-nm particles within a single droplet (*75*, *76*), shown for 11 particles tracked via fluorescence over a 30 s. (iii) Histogram of particle displacement for 200 particle trajectories over 30 s. For moving droplets, the NDs sample a larger part of the droplet volume (movie S1). (**E**) Co-encapsulation of cells with NDs. (i) Bright-field and (ii) fluorescence images showing yeast cells encapsulated along with 100 nm ND particles. Inset: Zoom into individual cells. See section S5 for ND targeting to these cells.

Our microfluidics platform, schematically depicted in Fig. 1A and photographed in Fig. 1A(i), features a device that produces phase-separated, monodisperse, droplets in a water-in-oil emulsion, varying in diameter in range 10 to 150 μm and in volume from 300 fl to 500 pl. Each chip supports multiple devices hosting diverse microfluidic structures and is made from polydimethyl siloxane (PDMS) via soft lithography and bonded to a glass cover slip [Fig. 1A(i)].

Droplets are formed by constricting an aqueous phase with oil channels [colored arrows in Fig. 1B(i)] using a fluid-focusing geometry at junction $J_{1}$, as depicted in Fig. 1A, through a ≈30 μm orifice. This creates a stable stream of uniformly spaced droplets shown in the bright-field image in Fig. 1B(i). Droplet size and formation rate are controlled by the orifice size and water/oil flow rates. We achieve droplet speeds in excess of 4 cm/s. The fabrication process ensures ≲1-μm error, guaranteeing high reproducibility across chips (section S2A).

In addition, the chips host a second junction $J_{2}$ (Fig. 1A), which enables oil injection to precisely control droplet spacing. This is demonstrated in Fig. 1B(ii), showing a spacing change from 154 to 360 μm. In a downstream region depicted in Fig. 1B(iii), droplet spacing remains constant and droplet flow is stable over several hours (section “High-stability quantum sensing in flow”). In this region (*a* in Fig. 1A), droplets are analyzed over a microscope objective and microwave (MW) coil for imaging and quantum sensing measurements before flowing through a circuitous path (*b* in Fig. 1A) that induces intradroplet mixing and extends the channel for downstream analysis of stationary droplets. Last, they are directed into a collection chamber (*c* in Fig. 1A), allowing for the simultaneous storage and further examination of over a hundred droplets (section S2B and movie S2).

Diamond particles are incorporated into droplets in flow using an aqueous suspension of carboxylated (hydrophilic) NDs, which host ∼1 to 3 parts per million (ppm) NV centers, as the dispersed phase. Zeta potential measurements (section S1) reveal a surface charge sufficient to confer hydrophilicity and colloidal stability. The fluorescent NDs are thereby completely encapsulated within the droplet, as shown in Fig. 1C. An inverted configuration, with NDs in oil droplets surrounded by an aqueous phase, can instead be achieved by coating ND surfaces with polymer chains. We showcase ND loading and droplet control through three movies available in the Supplementary Materials. They illustrate droplet motion in bright-field (movie S2) and fluorescence (movie S3) through various chip structures in Fig. 1A, highlighting rapid ND droplet formation, droplet spacing regulation via the dual focusing junctions $J_{1},J_{2}$, and ND mixing within flowing droplets (movie S4).

Fluorescence imaging allows visualization of microdroplets loaded with NDs of varying sizes (section S4A describes optical setup), as illustrated in Fig. 1C with examples of (i) 40, (ii) 100, and (iii) 3-μm-sized particles. The NDs feature a heterogeneous size distribution (±30 nm) characterized by DLS spectroscopy (see section S1). In Fig. 1C(i), the 40-nm NDs achieve a well-dispersed distribution within the droplet, occupying 0.01% of its volume with minimal evident aggregation and each droplet containing around 10^6^ particles. The 100-nm particles shown in Fig. 1C(ii) are brighter and remain well dispersed in solution and the 3-μm particles [Fig. 1C(iii)], while even brighter, tend to settle at the droplet bottom in stationary droplets, indicating a trade-off between fluorescence intensity and colloidal stability.

At the opposite limit, Fig. 1D(i) shows the encapsulation of a single 3-μm ND particle in a droplet. These large particles remain stationary in a static droplet, but can be induced to sample the droplet volume upon motion (movie S4). For smaller particles however, Brownian motion is much more pronounced, allowing the NDs to traverse large swathes of the droplet volume. In Fig. 1D(ii), we measure trajectories of individual 100-nm ND particles within a droplet over 30 s (see movie S1). Figure 1D(iii) presents a histogram of total displacement for these particles, based on tracking 200 ND trajectories for the same interval. They traverse distances >5 μm, several fold larger than their diameter. The long tail in the distribution points to anomalous diffusion reminiscent of Levy flight processes (*39*). Overall, these large excursions promote interaction with droplet-confined analytes.

Microdroplets can also serve as picoliter-scale containers capable of co-encapsulating entities (*36*). We demonstrate this in (Fig. 1E) by loading yeast cells (*Rhodosporidium toruloides*) and 100-nm NDs into a droplet. Bright-field and fluorescence images [Fig. 1E (i) and (ii)] highlight the yeast and diamond particles, respectively, with the inset in Fig. 1F(i) showing a single yeast cell. Diamond aggregation, influenced by ions in the yeast growth medium, can be managed by altering the medium or modifying the diamond surface functionality (*40*). In section S5, we show that the NDs can be targeted to the yeast cells via surface functionalization with Concanavalin-A, a protein with affinity to the cell surface.

### In-droplet chemical sensing by ODMR

#### Lock-in ODMR and challenges for low LOD sensing

**Fig. 2.**
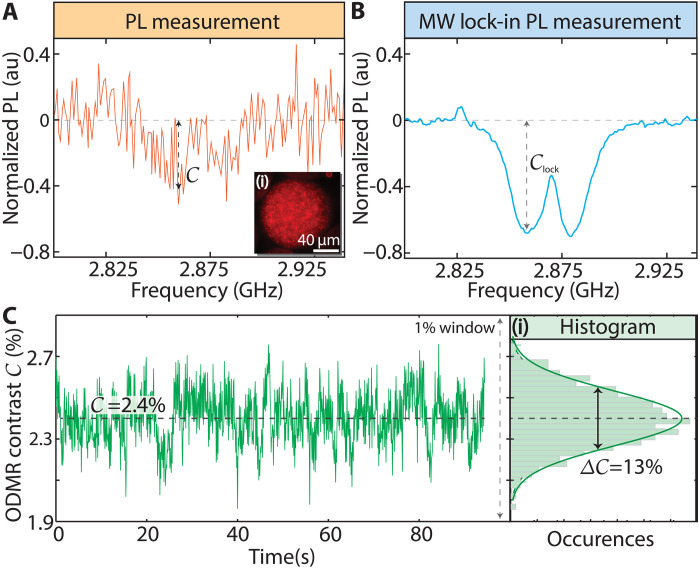
ODMR of ND particles in droplets. (**A**) Conventional ODMR measurement of 100-nm particles in a single ≈100-μm droplet at zero magnetic field. Inset (i): Droplet fluorescence image. Each point is averaged for 1 s with and without MWs. Contrast *C* is marked. Strong fluctuations arise from particle motion. (**B**) Enhanced ODMR using MW lock-in, using analog lock-in detector at modulation frequency $f_{\text{MW}}=1$ kHz. SNR improves 10-fold; contrast $C_{\text{lock}}$ is clearer. Strain-mediated dips around 2.87 GHz are visible. (**C**) ODMR contrast *C* relevant for chemical sensing obtained via normalizing lock-in signal in (B) at 2.866 GHz to simultaneously measured PL. Here, droplets are flowing, and data are sampled every 100 ms over 90 s. (i) Histogram of *C* data displays variations at the Δ*C* ≈ 13% level, highlighting the challenge for chemical sensing. Solid line is a Gaussian fit.

Chemical sensing uses the NV center’s electronic spin sensitivity to its environment and the ability to convert this into optical signals via its spin state–dependent fluorescence. This involves the measurement of an NV center optically detected magnetic resonance (ODMR) spectrum, where ND photoluminescence (PL) is monitored as MW excitation is swept in frequency across NV spin transitions (Fig. 2A). At Earth’s field, the PL exhibits a characteristic dip at ≈2.87 GHz, signifying a shift in spin population from the brighter $m_{s}=0$ state to the dimmer $m_{s}=\pm 1$ states on-resonance.

Figure 2A shows the first-reported ODMR measurements from ND particles in a single microdroplet, here held static [shown in Fig. 2A(i)]. PL here is at the subnanowatt level and is detected using a multipixel photon counter. These measurements are inherently noisy due to background, including PDMS autofluorescence (section S6), and fluctuations stemming from Brownian motion and particle re-orientation.

To enhance measurement signal-to-noise ratio (SNR), we use MW lock-in detection by amplitude modulating the applied MWs at $f_{\text{MW}}=1$ kHz. This lock-in frequency is chosen from a balance between rates of NV repumping and MW-driven population redistribution. The result, shown in Fig. 2B, is an order-of-magnitude increase in SNR making the strain splitting near 2.87 GHz clearly visible.

The ODMR contrast, marked *C* in Fig. 2A, quantifies the ODMR dip relative to off-resonance PL. At fixed laser and MW powers, *C* serves as a proxy for the NV electronic $T_{1}$, which is influenced by dipolar interactions with analytes in the droplet (*17*). Low limit of detection (LOD) sensing necessitates the ability to detect minute changes in *C* (*41*). This lock-in detection scheme effectively isolates diamond signals from noise and background, but for the purposes of analyte sensing across different populations of ND sensors, it is necessary to measure the ratio of ODMR signal to a baseline PL. While the lock-in strategy in Fig. 2B yields an increase in SNR, its contrast $C_{\text{lock}}$ remains susceptible to non–analyte specific factors like fluorescence variations from background and particle motion due to the absence of an off-resonance reference in this measurement. This poses challenges for chemical sensing.

A mitigating strategy involves continuously measuring NV PL and normalizing it to the resonant ODMR lock-in signal in Fig. 2B, creating a ratiometric measurement shown in Fig. 2C. In addition, measuring droplets in flow enables signal averaging over several droplets. Here, the ODMR signal from flowing droplets with MWs at 2.86 GHz over 90 s is normalized to the total PL measured at each instant. The normalized contrast, still referred to as *C* for convenience, in Fig. 2C has an average of 2.4% (dashed line); a 1% contrast window is shown here for clarity. Nonetheless, noise from PL measurements still affects *C*. Sensitive detection of analytes is limited by the ability to resolve small fluctuations about this baseline contrast *C*. Δ*C*, and by convention, we report it as a percentage of the base *C* level, rather than in absolute units. *C* itself varies based on sample and experiment conditions, but the percent error Δ*C* allows for a standardized comparison across different experiments. From a histogram of the data [Fig. 2C(i)], we estimate Δ*C* ≈ 13% in this case, setting a bound on the quantitative sensing at low LOD.

#### Double lock-in quantum sensing in droplets

**Fig. 3.**
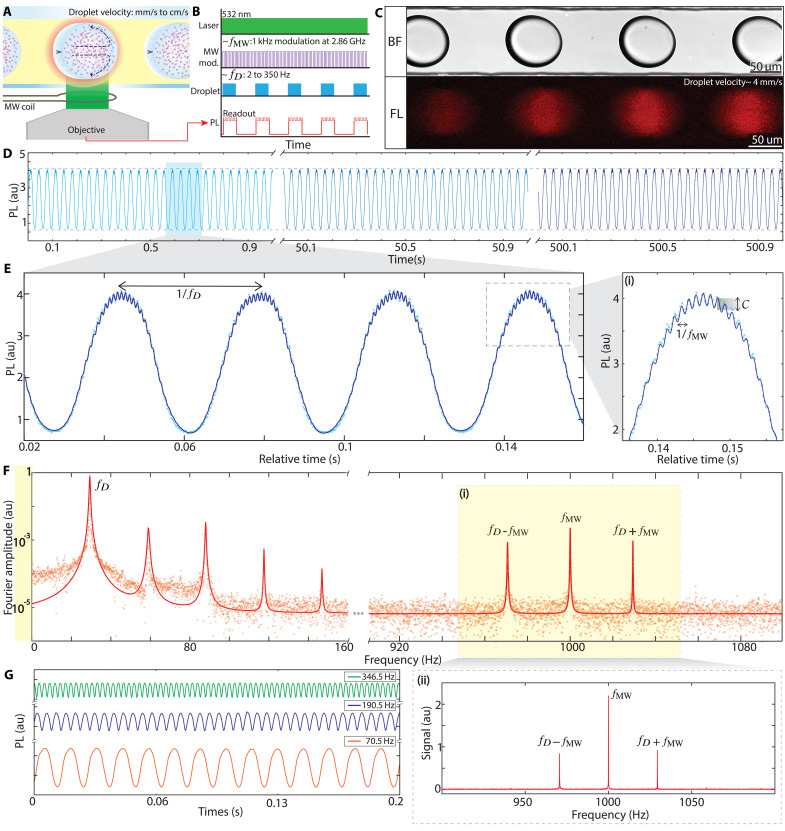
Droplet double lock-in detection. (**A**) Schematic and (**B**) protocol: Droplets hosting NDs flow through the analysis region where they are illuminated by continuous 532-nm illumination, and MW excitation at the ODMR resonance. Two modulations are imposed upon the PL (red line) by droplet flow at *f*_*D*_, and MWs at *f*_MW_. Mixing is illustrated by the arrows. (**C**) Bright-field and fluorescence images of droplets containing 100-nm NDs flowing at *f*_*D*_ ≈ 30 droplets per second. (**D**) Long-time PL from flowing droplets captured over a 8.3-min period. Three panels show representative 1-s windows. Dashed horizontal lines indicate intensity limits. (**E**) Double modulation imprinted into PL. Representative 140 ms (≈4 droplet) segment of the PL trace in (D). PL modulates at *f*_*D*_ due to successive droplets entering and leaving the field of view. (i) Smaller ODMR modulation reflected by gray shading at *f*_MW_ is observable in the zoom-in. Blue points represent the data, and the solid line is a fit to Eq. 1. (**F**) Fourier transform intensity of PL collected over *t* = 15 s displayed on a logarithmic scale. Points are data and solid lines are Lorentzian fits. Droplet modulation generates a sharp peak $F\left(f_{D}\right)$ at $f_{D}$ and its harmonics, stemming from the square wave–like droplet profile. The peak at zero frequency is excluded for simplicity. Sharp peaks arise at the MW modulation, $f_{\text{MW}}=1$ kHz, while a combination with flow leads to peaks at $f_{\text{MW}}\pm f_{D}$ (yellow shaded regions). Narrow Fourier linewidths are evident for all peaks (see Fig. 4B). (ii) Linear scale FT: Data in yellow shaded region in main panel are plotted on a linear scale highlighting narrow linewidth and high SNR. (**G**) Tunability of droplet modulation. PL traces for three example cases in 200-ms windows. Highest frequency $f_{D}=346.5$ Hz corresponds to sampling >10^6^ droplets per hour.

To improve measurement precision and render ODMR contrast immune to background fluctuations, we use a strategy using droplet flow, as illustrated in Fig. 3A. Droplets, uniform in size and ND content, move at a controlled velocity (*v*) and sequentially enter the analysis region, where an optical spot, roughly equal to the droplet diameter, illuminates them. The spacing between droplets is ${\mathrm{vf}}_{D}^{-1}$, where $f_{D}$ is the rate at which droplets are analyzed in region *a* in Fig. 1A, and *v* ranges from 1 mm/s to 4 cm/s in our experiments.

Simultaneously with flow, droplets are subjected to MWs at 2.866 GHz, and amplitude modulated at $f_{\text{MW}}=1$ kHz (Fig. 3B) under continuous laser illumination, introducing two distinct modulations to the PL: $f_{D}$ and $f_{\text{MW}}$ (Fig. 3B). We make use of this arrangement to implement a double lock-in measurement as has been demonstrated in other contexts (*42*). By arranging $f_{\text{MW}}\gg f_{D}$, each droplet’s signal contains multiple MW modulation cycles, and lock-in detection at the multiple generated frequencies helps to filter out background noise.

**Fig. 4.**
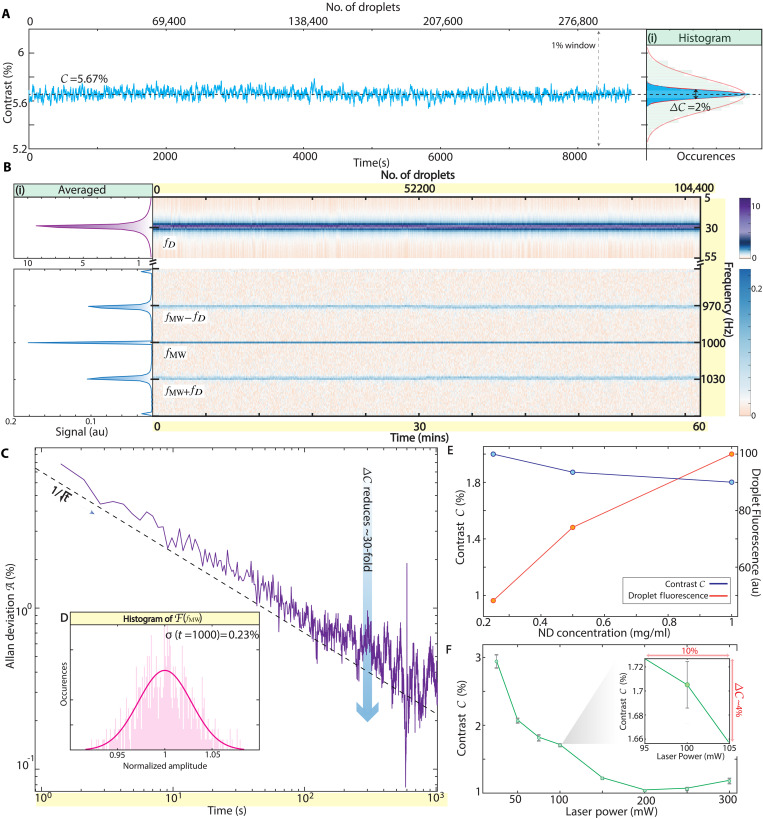
High stability in-flow droplet measurement. (**A**) Long-time high-precision measurement of ODMR contrast *C* following Eq. 2 over *T* ≈ 2.5 hour period, encompassing >290,000 droplets (upper axis), demonstrating stability. Data are sampled every ≈1 s. (i) Inset: Blue bars show histogram of the measured contrast *C* in (A). Solid line is a Gaussian fit; from the linewidth we estimate the percent error ΔC=2% of the mean contrast (dashed line). Green histogram from Fig. 2C(ii) is overlaid for reference, highlighting narrowing via double lock-in scheme. (**B**) Spectrogram of the Fourier peaks in frequency bands around $f_{D}$ and $f_{\text{MW}}$ measured over 1 hour. Data are presented over successive 700-ms windows (corresponding to 20 droplets), for a total of 104,000 droplets (upper axis). Upper and lower windows span 50 and 120 Hz, respectively. Colors indicate FT intensities F(f) of PL in the two frequency bands. (i) Left panels: Integrated intensity of the spectrogram data plotted against frequency. Narrow peaks indicate high stability over the entire period. (**C**) Allan deviation A(t) shown for 1000 s of data in (B). A(t) follows $\propto 1/\sqrt{t}$ trend (dashed line) for the entire period, highlighting remarkable stability. Percent error ΔC reduces over 30-fold as a result. (**D**) Bounding ND variation per droplet. Histogram of the intensity of $F(f_{\text{MW}})$ peak from spectrogram in (B), measured over 7-s bins. Solid line is a Gaussian fit. Extrapolating to 1000 s (main panel), we estimate inter droplet ND variation <0.23%. This corresponds to ≲2300 particle variation over ≈1M NDs per droplet. (**E** and **F**) Compensation of experimental variations via Eq. 2. Variation of ODMR contrast *C* with ND concentration (E) and with laser power (F). Red line shows measured droplet fluorescence, while blue line shows corresponding *C*. Inset: The operational regime for our experiments at ≈100 mW.

Since the method exploits droplet flow, we highlight some of its features. Fig. 3C illustrates bright-field and fluorescence images of diamond-filled droplets in motion, complementing the stationary images in Fig. 1C (see also section S8 and movie S5). The flow exhibits remarkable regular modulation at $f_{D}$ due to droplet monodispersity, as evidenced by Fig. 3D, which displays measured PL over 500 s and >10^4^ droplets. Individual 1-s windows are shown here, with dashed rails highlighting droplet stability (see also Fig. 4).

Figure 3E zooms into a representative 140-ms window, showing modulation in the PL from both droplet flow and from the MWs. Solid lines fit the data. The distinct timescales of both droplet and MW modulations are evident. This is clearer in the inset, Fig. 3E(i), which focuses on a 30-ms window, illustrating oscillations spaced by $f_{\text{MW}}^{-1}$ (1 ms), consistent with the ODMR contrast in Fig. 2C.

The double lock-in measurement is detailed in the Materials and Methods and summarized here. Figure 3F displays the Fourier transform amplitude F of the PL time series from Fig. 3E, measured over 15 s of droplet flow, after subtracting its mean value. Data are shown on a logarithmic scale for clarity. It features a distinct Fourier peak at $f_{D}=29$ Hz with a narrow linewidth ($\Delta f_{D}\approx 1$ Hz), reflecting minimal drift in flow rates (see Fig. 4). Solid lines are Lorentzian fits. Square-like modulation leads to secondary harmonics at multiples of $f_{D}$, while background signal and autofluorescence occur near zero frequency and can be excluded.

Expanding Eq. 1 (Materials and Methods) reveals frequency bands linked to MW modulation and their combinations with flow, at $f_{\text{MW}}$ and $f_{\text{MW}}\pm f_{D}$. This is shown in the yellow shaded region, Fig. 3F(i). The peak intensities here are ∼1% that of the droplet modulation, reflecting the contrast C. Figure 3F(ii) shows the same frequency window in a linear scale for clarity. The ODMR contrast C(t) can now be calculated from the ratio of FT peak intensities at the MW and droplet frequencies. In this calculation, it is important to account for fluctuations in droplet frequency over time (see Materials and Methods for details).

Figure 3F also highlights that the noise profile diminishes $\underset{\sim }{\propto }1/f$, suggesting that higher droplet rates could lead to lower noise while enabling greater analysis throughput. To demonstrate the versatility and control over droplet modulation in our experiments, Fig. 3G shows smoothed PL profiles at various $f_{D}$ rates. At the upper limit ($f_{D}=346.5$ Hz), more than a million droplets can be analyzed per hour (Fig. 4C), the droplets flowing here at a rapid velocity, v> 5 cm/s (see movie S5).

Figure 4A depicts the result of Eq. 2 applied to 40-nm particles in droplets flowing at $f_{D}=34$ Hz, measured over a long period ($T=10^{4}$ s). Data are sampled every 100 ms (corresponding to roughly three droplets), and the top axis quantifies the droplet count. The ODMR contrast, C=5.6%, is marked by the dashed horizontal line in Fig. 4A. Data are displayed on an identical 1% contrast window to draw a comparison to Fig. 2C. The right panel of Fig. 4A(i) shows this as a histogram, overlaid with the analogous histogram from Fig. 2C using the analog lock-in for clarity. The histogram linewidth in the case of Fig. 4A narrows substantially to ΔC=2%, highlighting the enhanced measurement precision. Figure 4A also illustrates the inherent stability in the measurement of contrast C, here over more than 2 hours and 250,000 droplets.

#### High-stability quantum sensing in flow

To delve deeper into the observed stability in Fig. 4A, we analyze the data as a time-domain spectrogram in Fig. 4B. This entails taking a Fourier transform of the PL over small windows Δt=0.7 s, equivalent to 20 droplets, for an hour, and tracking the resulting spectra over 104,400 droplets (top axis). Colors in Fig. 4B represent Fourier spectral intensity, and the vertical position indicates frequency. We focus on frequency windows near $f_{D}$ and $f_{\text{MW}}$, using two distinct color bars for clarity. The left panel of Fig. 4B(i) shows the integrated signal across these bands. The narrow linewidths, evident over this extended period, reflect the system stability.

Another perspective on the stability is provided through an Allan deviation analysis, applied to the data in Fig. 4B and depicted in Fig. 4C. Allan deviation, A(t), provides insight into how measurement precision of C (ultimately related to analyte LOD) may improve with longer averaging time *t* or increasing number of droplets. Remarkably, our experimentally measured A(t) closely aligns with the theoretically expected $\propto 1/\sqrt{t}$ trend (dashed line) beyond 10^3^ s of averaging, yielding a >30-fold reduction in ΔC (blue arrow in Fig. 4C). At the lowest point, this corresponds to detecting PL changes to a few-hundredths of a percent. Figure 4C marks a notable improvement over previous studies (*35*, *43*–*45*), where equivalent $\propto 1/\sqrt{t}$ scaling is challenging to obtain, and only achievable through highly sophisticated compensation strategies. We attribute this enhanced stability to the immunity of the confined aqueous droplet volume to temperature drifts, their exposure to laser illumination only for short instants ($f_{D}^{-1}∼33$ ms), and compensation for laser power fluctuations and ND loading variations by the ratiometric scheme in Eq. 2.

Variability in FT intensities of the band centered at *f*_MW_ provides a convenient means to estimate an upper bound on the interdroplet ND variation. Figure 4D shows histogram of the $F(f_{\text{MW}},t_{i})$ amplitude binned every 7 s (∼200 droplets). When extrapolated to 10^3^s following a $t^{-1/2}$ scaling, we obtain an ND variation across droplets lower than 0.23%. In absolute terms, this corresponds to a very low droplet-to-droplet variation of ≲2300 NDs over the base level of ≈1M NDs per droplet.

Now, exploring the impact of potential drift mechanisms, in Fig. 4 (E and F), we assess how laser power and ND concentration variations affect ODMR contrast *C*. First, in Fig. 4E, we adjust ND concentration over a large range in the flowing droplets, using a 10-way valve (section S2C) to load successive samples containing different concentrations while maintaining fixed laser and MW powers. Red points in Fig. 4E show the resulting change in droplet PL, while blue points show the measured ODMR contrast *C* in percent units. Despite a large, ∼500% increase in ND concentration, ODMR contrast variation is less than Δ*C* ∼ 11%. Given that interdroplet ND concentration variation is <0.23% (Fig. 4D), ND number variations minimally affect contrast.

Increasing laser power, with MW power held constant (Fig. 4F) affects *C* due to an interplay of NV center repolarization and MW-driven population shifts. However, as Fig. 4F indicates, at our operational power (∼100 mW), a 10% laser power variation alters ODMR contrast by only about Δ*C* = 4%. In reality, laser drift is under 1%, and thus leads to minimal effects on Δ*C*. Overall, Fig. 4 (E and F) underscores the method’s resilience to common experimental fluctuations.

We comment finally that the double lock-in scheme could be performed through alternate approaches. MW modulation could be replaced by magnetic modulation, with potential advantages of a higher modulation depth (>10%) (*46*–*48*). However, it lacks the precise frequency control and long-term stability of MW lock-in. The latter also benefits from lower 1/*f* noise due to its higher operating frequency. While laser modulation, such as with an optical chopper, is a possible alternate method to $f_{D}$ droplet modulation, droplet flow, as introduced here, has distinct advantages: (i) immunity to autofluorescence (section S6), (ii) averaging effects across droplets to reduce heterogeneity, (iii) suitability for high-throughput analysis in a flowing geometry with (iv) stable $f_{D}$ modulation for several hours, and (v) improved thermal stability of flowing versus static droplets, ensuring reliability over long-time analyses. Some of these approaches, or combinations could also be implemented in a wide-field imaging configuration, averaging over many stationary droplets, although such an approach would introduce limitations in autofluorescence mitigation, stability, and instrumentation requirements.

#### Detection of paramagnetic species in flow

**Fig. 5.**
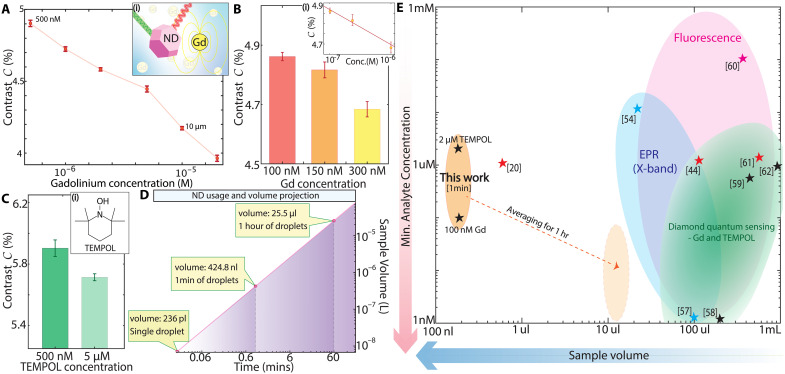
Sensing paramagnetic species in flowing microdroplets. (**A**) Gd^3+^ ion detection. ODMR contrast *C* as function of Gd^3+^ concentration measured in ≈50 μm droplets flowing at $f_{D}∼$ 40 Hz. Datapoints are red circles; error bars reflect Δ*C* for 4-min measurements. Inset: Schematic of sensing. Spin noise from ${Gd}^{3+}$ ions affects NV *T*_1_, and converts to measurable change in ODMR contrast *C*. (**B**) Gadolinium sensitivity. Results of separate experiment measuring a lower range of ${Gd}^{3^{+}}$ concentration. Inset (i)*:* Data plotted against a log scale in concentration. We estimate a LOD ≈100 nM. (**C**) TEMPOL sensing. Similar measurements for two concentrations of TEMPOL in flowing ≈50 μm droplets over 1 min each. We estimate a LOD <2 μM. (**D**) Volume scaling for in-droplet sensing shown on a logarithmic scale. Marked points correspond to a single droplet, 1 min, and 1 hour of measurements, assuming flow at $f_{D}=30$ Hz. (**E**) Landscape of chemical quantum sensing techniques. Comparison of related methods (diamond sensing, EPR, and fluorescence) for radical/paramagnetic analyte concentration on axes corresponding to sample volume required and lowest detectable concentration reported. Ideal sensing constitutes bottom-left region of plot (arrows). Stars are derived from specific references; shaded regions illustrate approximate sensing boundaries. Our work is shown by the orange region. Red stars represent single crystal based sensing and black stars represent ND-based sensing. Light orange region shows projected improvement from averaging to 10^3^ s (Fig. 4C).

Leveraging the enhanced precision above, we detect chemical analytes in flowing droplets, starting with Gadolinium (Gd^3+^) ions in GdCl_3_ hexahydrate as a model system. The sensing mechanism, depicted in Fig. 5A(i) inset, relies on changes in the NV center *T*_1_ relaxation time from spin noise of paramagnetic species, affecting the $m_{s}=\pm 1$ population and, thus, ODMR contrast. This effect is concentration dependent, allowing for quantitative analyte estimation. Enhanced precision via Figs. 3 and 4 enables the detection of subtle contrast changes, greatly improving LODs.

In Fig. 5A, we demonstrate this through Gd^3+^ concentration titration in droplets, with each sample averaged for ≈4 min. Samples are automatically loaded using a 10-way valve (section S2C) controlled by a customized program. Figure 5A highlights the dynamic range over which this system can detect gadolinium ions, ranging from 500 nM to 20 μM, above which the ionic strength of the solution causes aggregation of our carboxylated diamond particles. We further investigate the sensitivity at the low end in a separate experiment loading 100, 150, and 300 nM samples in Fig. 5B. We achieve a LOD of 100 nM over 2 min of averaging.

While Gd^3+^ has spin S=7/2, most practical applications target single unpaired electronic spins (S=1/2). We thus use 4-hydroxy-2,2,6,6-tetramethylpiperidine-1-oxyl (TEMPOL) as a second model [Fig. 5C(i)]. TEMPOL is a stable radical probe that could serve as a proxy for other paramagnetic analytes, including ROS, relevant to, for instance, metabolic studies in cells. Figure 5C shows the ability to differentiate between 500 nM and 5 μM concentrations. We estimate an LOD of 2 μM over 1 min of measurement.

Figure 5D highlights how our droplet-based sensing requires a very small quantity of NDs. The red line shows sample volume used for varying measurement times, 1 droplet, 1 min of averaging, up to 1 hour. Such low volumes result in minimal required reagent, using <$1 for 1000 s of analysis involving $∼3\times 10^{4}$ droplets. This benefits also from the NDs’ native hydrophilicity (*49*) (section S1), which negates the need for surface treatments. Our method therefore not only reduces costs dramatically compared to traditional single-crystal diamond approaches requiring costly growth and surface modifications but also enhances portability. Combined with the data from Fig. 4, this showcases the capability for long-term, stable, and precise analyses at reduced costs.

Last, Fig. 5E shows an overview of the sensing technology landscape, identifying the niche filled by droplet-based quantum sensing. We focus attention here on gadolinium and TEMPOL detection, and comparisons to widely used methods for ROS and spin trap detection. We assess electron paramagnetic resonance (EPR) (*50*–*52*), fluorescence, and NDs-based sensing across reported LODs and analyte sample volume (Fig. 5E). Table S1 provides more detail contrasting diamond-based quantum sensing methods. An ideal chemical sensing platform would occupy the bottom left corner of this plot. Although direct comparisons are challenging due to the diversity of implementations in each technique, we show regions of applicability of each method (shaded regions in Fig. 5E) and representative references (marked points) (*20*, *41*, *50*, *53*–*58*). Droplet-based sensing as described here occupies the orange region and is estimated to move along the orange-dashed arrow, with 1 hour of averaging following Fig. 4C. Figure 5E illustrates that our method already provides notable improvements over existing technologies.

## DISCUSSION

Our work combines droplet microfluidics with quantum sensing and introduces many innovations, including (i) deploying ND quantum sensors within droplets, using confinement and flow to (ii) facilitate continuous analysis with high precision, capable of detecting contrast changes by ΔC≈2%, (iii) with high stability across multiple hours and hundreds of thousands of droplets, (iv) all while using minuscule sensor volumes and entailing incredibly low ND costs. Looking forward, the platform technology introduced here anticipates many interesting directions.

First, the use of ND-loaded droplets opens possibilities for moving beyond traditional single-crystal diamond sensors to those that closely interact with analyte molecules. Mixing within the droplets averages out particle heterogeneity, and averaging over many droplets is simple, rapid, and improves sensitivity (Fig. 4C). The mobility afforded by droplet confinement introduces versatility in sensor manipulation, such as via droplet sorting, splitting, and collisions (*59*), and precise placement over target samples. Using electrostatic forces, charged droplets can be dynamically organized (*60*), forming consistent quantum sensor ensembles that could enable the creation of rearrangeable three-dimensional sensor arrays. We also foresee the development of an “ND particle sorter” capable of isolating high-quality diamond particles from random populations based on desired characteristics, such as NV center $T_{2}$ times, streamlining the production of quantum materials for sensing.

Our in-flow analysis method, with its precision, stability, and low-volume capabilities, enables high-throughput, high-sensitivity chemical assays (*61*). These assays can be conducted serially using single droplets or in a wide-field setting using a lock-in camera (*44*, *62*–*64*). They would be ideal for analysis in amplification-free scenarios or in optically turbid media (e.g., blood), eliminating the need for washing steps, and opening applications in bioengineering, trace pathogen detection for diagnostics, and chemical reaction monitoring (*65*). Moreover, the assay devices can be rendered compact and portable, thanks to the remarkably low cost of the ND sensors (Fig. 5C), even lower than the reagents for droplet generation, combined with high stability (Fig. 4C), and robustness to laser power drifts (Fig. 4F). The latter suggests feasibility of using low-cost diode lasers, suggesting compact, field-deployable devices.

Using droplets for cell confinement heralds new directions in high-throughput single-cell analysis. This technique is particularly promising for methods akin to flow cytometry (*66*), focusing on cellular metabolism through detecting intracellular paramagnetic ROS (*67*). We anticipate “quantum-enhanced” flow cytometry, introducing additional dimensions such as cell-localized $T_{1}$ relaxometry, providing insights into single-cell metabolomics alongside traditional cell morphology information.

When combined with real-time sampling, this could also enable unprecedented intracellular measurements directly from bioreactors. By integrating cellular lysis buffers and reagents within droplets or through droplet collisions, we imagine a rapid cellular analysis method aimed at precisely controlling bioreactor conditions to achieve optimal outcomes.

More fundamentally, our approach unlocks possibilities for in situ chemical imaging and kinetic analysis within the confined volume of a microdroplet (*60*). ND particle tracking (Fig. 1E and movie S1) enables new methods for real-space imaging of diffusion in microconfined environments, including turbid settings, opening new perspectives in chemical and biological systems.

The Allan deviation depicted in Fig. 4C highlights measurement stability among best reported in literature, but achieved with notable ease of operation. This suggests ND-filled flowing droplets could serve as a promising platform for applications in bulk magnetometry (*68*) and as accelerometers and rotation sensors (*69*, *70*). Last, we note other properties of NDs not used in this study but fully compatible with being deployed in flowing droplets. These include the ability to hyperpolarize ${}^{13}C$ nuclear spins in NDs optically via NV centers (*71*), and exploit their multiple-second-long transverse lifetimes $T\prime_{2}$ (*72*) to construct movable sensors, high-field magnetometers, and NMR sensors within droplets (*73*).

## MATERIALS AND METHODS

### Materials

Diamond particles of all sizes are from Adamas Nanotechnologies. Unless otherwise stated particles are carboxyl terminated and implanted with approximately 2-ppm NV centers. Particle size and surface charge characterization data from dynamic light scattering and zeta potential measurements are detailed in section S1.

### Microfluidics and microdroplet production

All microfluidics experiments use custom devices, designed in house and fabricated in the U.C. Berkeley Biomolecular Nanotechnology Center. Chips are made using a silicon master wafer for photolithography to produce PDMS chips that are plasma bonded to glass coverslips. Detailed fabrication procedures are discussed in section S2.

To generate microdroplets, pressure controlling pumps (Fluigent FlowEZ) pressurize reagent vessels filled with a dispersed phase (aqueous in our case) and a continuous phase (2% SPAN80 (Sigma-Aldrich) surfactant in mineral oil) and fitted with pressure caps to deliver fluid to the devices. After inserting inlet tubing and outlet tubing (1/32″ outer diameter) into the device, pressure applied to the water and oil channels at a ratio of approximately 1:2 (w:o) creates droplet emulsions. We adjust set points to optimize for desired flow rate, droplet size and droplet spacing. Titration experiments use a 10-way valve (Fluigent m-switch, see section S2C) to controllably deliver successive samples to the device.

### Imaging and ODMR

Fluorescence images and ODMR measurements were performed on a custom-built microscope system that has been reported previously (*74*) and is described in section S4A. A schematic of the setup is shown in fig. S7. Some wide-field images were collected using a Nikon Ti2U microscope with the same camera (Teledyne Kinetix) at either 4×, 10×, 20×, or 40× magnification. Diamond fluorescence is collected using CYT5-HQY filter cube (excitation/emission, 620/710 nm) or mcherry filter cube (excitation/emission, ≈540/600 nm).

MW excitation is generated using a MW synthesizer (HP 8664A) outputting 0 dBm into a MW switch which controls whether the MW are passed to the sample or “off” (Minicircuits ZYSW-2-50DR). A 16-W amplifier (Minicircuits ZHL-16W-43-S+) increases the power input into a 100-W amplifier (empower BBM4A6AK5), the output of which passes through a circulator (Pasternack, PE83CR1004) before being delivered to the sample using a circular loop antenna placed between the objective and the sample such that the laser passes through the center of the loop. MWs are modulated at a chosen rate (generally 1 KHz) by using the reference output of a lock-in amplifier (Stanford Research SR830) to drive the MW switch.

The fluorescence signal is detected by a multipixel photon counter (Hamamatsu C14452) operating in Geiger mode to generate a photovoltage which is directed both to a data acquisition card (NI DAQ 6215) and to the input of the lock-in amplifier. Lock-in settings like sensitivity, lock-in time constant, and dynamic reserve are chosen to maximize SNR and minimize measurement time and are dependent on signal magnitude and the timescale of signal changes that need to be detected.

For measurements using the lock-in amplifier a customized LabView program scans MW frequency while measuring lock-in and fluorescence signal or measures signal over time at a set MW frequency as droplets pass through the field of view. For double lock-in measurements fluorescence photovoltage is measured directly using the analog channels of a data acquisition card.

### Double lock-in measurement details

The PL double modulation seen in Fig. 3 can be expressed as the functional form

$$S\left(t\right)=\left[m\left(t\right)+g\left(t\right)\text{cos}\left(2\pi f_{D}t\right)\right]\cdot \left[1-C\left(t\right)\text{cos}\left(2\pi f_{\text{MW}}t+\phi \right)\right]+b\left(t\right)$$ (1)

where *g*(*t*) represents the droplet modulation profile influenced by droplet shape and separation. Microfluidic control enables adjustable profiles, from sinusoidal to square-like (section S3). C represents the ODMR contrast, with time dependence included to account for possible long-term drift across numerous droplets. *m*(*t*) reflects the baseline ND contribution to PL, becoming more prominent as interdroplet spacing decreases [and controllable via junction $J_{2}$, see Fig. 1B (ii)]. Last, *b*(*t*) captures PL noise from non-ND contributions.

The droplet number–normalized ODMR contrast, calculated from the dual modulation profile within fixed time windows Δt, can be expressed as

$$C\left(t_{i}\right)=\frac{F\left(f_{\text{MW}},t_{i}\right)+F\left(f_{\text{MW}}+f_{D},t_{i}\right)+F\left(f_{\text{MW}}-f_{D},t_{i}\right)}{F\left(f_{D},t_{i}\right)}\cdot \frac{1}{f_{D}\left(t_{i}\right)}$$ (2)

Here, $F(f,t_{i})$ denotes the Fourier intensity at frequency *f* for a time interval bounded by $t_{i}$ and $t_{i}-\Delta t$, averaging the PL over several droplets. Dividing the MW-associated FT peak intensities by the droplet frequency FT peak intensity yields a ratiometric ODMR contrast, factoring in the droplet PL. Equation 2 also includes a normalization proportional to droplet frequency to counteract minor frequency drifts that affect the baseline ND-dependent PL [*m*(*t*)], providing a consistent contrast metric irrespective of droplet flow rate.

## Supplementary Materials

### The PDF file includes:

Sections S1 to S12 Figs. S1 to S10 Table S1 Legends for movies S1 to S6 References
