# Supplementary material for: High-precision chemical quantum sensing in flowing monodisperse microdroplets
Source: Sci Adv. 2024 Dec 11;10(50):eadp4033. doi: 10.1126/sciadv.adp4033 (PMC11633744; doi:10.1126/sciadv.adp4033)
Supplement: Supplementary file 1 — Sections S1 to S12 Figs. S1 to S10 Table S1 Legends for movies S1 to S6 References [file sciadv.adp4033_sm.pdf]

Science Advances
AAAS

Supplementary Materials for

High-precision chemical quantum sensing in flowing monodisperse microdroplets

Adrisha Sarkar *et al.*

Corresponding author: Ashok Ajoy, ashokaj@berkeley.edu

Sci. Adv. **10**, eadp4033 (2024)
DOI: 10.1126/sciadv.adp4033

The PDF file includes:

Sections S1 to S12
Figs. S1 to S10
Table S1
Legends for movies S1 to S6
References

Other Supplementary Material for this manuscript includes the following:

Movies S1 to S6

S1. NANODIAMOND SIZE AND CHARGE

Particle sizes and charges are determined by the manufacturer using dynamic light scattering (DLS) with zeta potential. Results of particle characterization are shown in Fig. S1 showing hydrodynamic diameter (A) and Zeta-potential (B) for the 40 nm and 100 nm particles used in our experiments. The DLS volume average (A) shown gives an indication of the average size and population heterogeneity, and is skewed toward higher values as large particles scatter more than small. Number mean (d_n)given as the inset is indicative of the average size of the population of particles. The particles represent a distribution of sizes with number mean 37 nm and 107 nm for 40 nm. The width of the peak is indicative of variation in sizes that demonstrates particle-to-particle variability, especially in the smaller sized particles which have a FWHM$\approx$ 30 nm.

Both sets of particles have robust colloidal stability owing to their zeta potential (ζ). A measure of the surface charge of nanoparticles in solution, ζ gives information about the colloidal stability of the NDs. In general, nanoparticles with zeta potential less than -30 mV (or greater than +30 mV) will remain suspended in aqueous solution. (*79*) This is largely due to electrostatic repulsion between particles ensuring they stay separated by enough distance to avoid aggregation. Fig. S1B shows that the NDs have negative surface charge: -37 mV for the 40 nm particles and -56 mV for the 100 nm particles, which is sufficient to remain well dispersed in our aqueous samples.

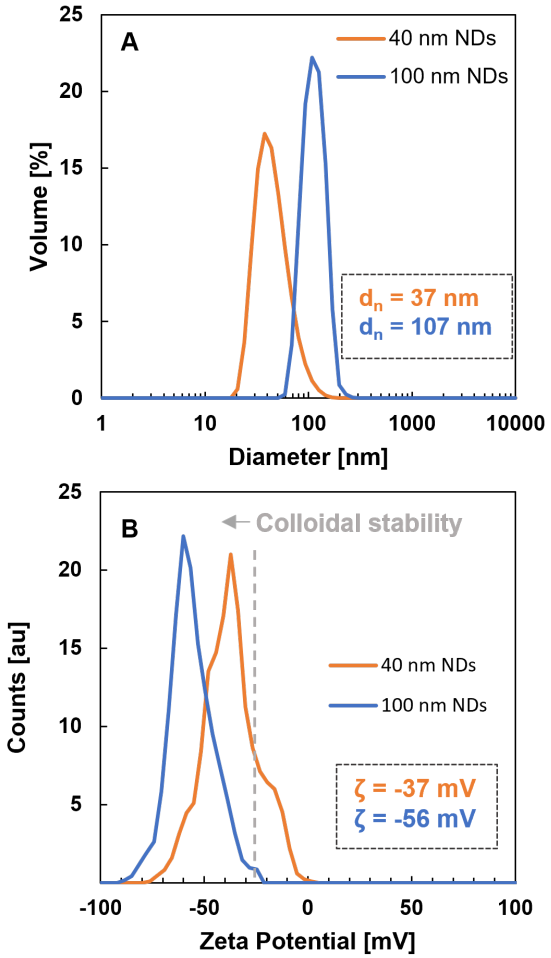

Fig. S1: **Nanodiamond DLS and Zeta potential measurements.** Size and surface charge characterization for the 40 nm and 100 nm particles used in our experiments. Dynamic Light Scattering (DLS) plots (A) show histograms of the measurement of hydrodynamic diameter for the population of particles measured. Volume average plot are shown, indicating the proportion of the volume of particles comprised of a given diameter, while number mean (average sizes of all particles) is given as an inset. Zeta potential measurements (B) shows histograms of the measured surface charge from a population of particles, along with the reported value (inset). Dashed line represents roughly the deliniation between colloidally stable and not.

S2. MICROFLUIDICS DETAILS

A. Device fabrication

Silicon Master wafer

A 4-inch silicon wafer is cleaned thoroughly with an acetone wash followed by an IPA wash, then dried with nitrogen and placed on a 150°C hotplate for 10 min. This wafer is then centered in a spin coater and vacuum is used to hold the wafer in place. A layer of photoresist (SU8-2025) is spin-coated to the wafer, under conditions recommended by the SU8 datasheet and spin coater specifications, to produce a 40um film thickness. About 4ml of the photoresist is poured onto the wafer and spun under the appropriate conditions (500 rpm for 10s followed by 2000 rpm for 30s in our apparatus). After removing the wafer from the spin coater it is pre-baked on a hotplate at 65°C for 3 min and 95°C for 6 min. A mylar photomask with the microfluidic device design is used to UV expose and cure the photoresist. After cooling to room temperature, the wafer is placed in a mask aligner with the photomask on top of it, ink side down. The mask aligner is closed and clamped down ensuring the best possible contact for the wafer and photomask. Based on the UV power delivered by the mask aligner we calculate the exposure time:

Exposure time = (Exposure Dosage) / (Power Intensity) * (Scaling Factor)

For 40 μm channel height for 160 mJ/cm^2 energy, we expose for 20 s at 10 mW/cm^2. Post exposure, the wafer is baked on the hotplate for 1 min at 65 °C and then 6 min at 95 °C. Once the wafer has cooled to room temperature it is submerged in an SU8 developer bath with agitation for 7.5 min then rinsed with isopropyl alcohol. If white streaks are noticed, the wafer is underdeveloped and is re-submerged in developer in 20 s increments till no streaks are visible. Once no SU8 residue is present on the wafer, it is rinsed with isopropyl alcohol and deionized water then dried. The wafer is finally hard baked at 150 °C for 10 min. Stylus profilometry and light microscopy are used to assess feature sizes and integrity.

Chip fabrication

To create the PDMS on glass microfluidic device we begin by creating a PDMS cast from the master wafer. A Sylgard 216 A+B Clear Silicon compound consisting of a PDMS pre-polymer (A) and curing agent (B) which are vigorously mixed together at a 10:1 ratio. 10 mg of PDMS pre-polymer and curing agent per square inch provided an adequate thickness (~5mm) for the microfluidics device. The total volume of PDMS prepared can be adjusted to suit the device thickness desired. The mixed PDMS is then left to degas in a vacuum chamber for 30-60 min until all gas bubbles disappear. This uncured PDMS is poured onto the

master mold. The master mold is laid flat, pattern side up in a petri dish and the PDMS is poured on top then baked in a 65 °C oven for at least 6 hr and for up to 12 hr. Once removed from the oven and cooled, the desired PDMS portions for each chip are cut out carefully using an Xacto knife. The inlet ports for the microfluidics devices are created by punching vertical holes with a biopsy punch compatible with the inlet/outlet tubing diameter. To remove particulate matter and dust off the PDMS surface, scotch tape is used to clean and protect the PDMS.

Bonding to glass

A glass coverslip, is cleaned and prepare by wiping with a lint-free wipe, rinsing for 1 minute with Milli-Q water, and manually scrubbing all surfaces and edges. This is followed by an acetone and IPA wash and scrub. To remove chemical residues a final Milli-Q water wash is done without touching the main face of the glass. The coverslip is dried using compressed air and checked for marks or spots. If any such issues are noted the glass must be re-cleaned or a new coverslip must be prepped. It is important that the coverslip chosen for bonding to the PDMS is spotless.

The final PDMS-on-glass device is assembled using oxygen plasma bonding. Once the bonding chamber is initialized, the glass coverslip and PDMS piece are inserted into the chamber, both bonding face side up. The vacuum pumps are engaged to remove air until stabilized ($\lesssim$0.32 Torr). Oxygen gas is then released into the chamber until pressure re-stabilizes ($\approx$0.4 Torr). The radio-frequency (RF) power is turned on to deliver 24 W UV light for 24 s. The RF, oxygen and vacuum are disengaged to allow access to the bonding chamber. Immediately, the PDMS is touched to the glass and gently pressed to facilitate the bonding via the reactive oxidative species deposited. The device is left for >12 hrs in a 65 °C oven before use.

B. Device Features

We design varying photolithography masks to form structures like those shown in Fig. S2 to produce different droplet outcomes.

Fluid focusing junction

As discussed in the main text, we use a fluid focusing junction (see Fig. S2A) to form droplets at a constant size, and rate. We have flexibility in the speed and size of droplet production with control of the relative flow rates of the aqueous and oil phases entering the junction. Microfluidic device design affords an additional layer of control over these factors.

Droplet separator junction

We control the inter-droplet spacing via the addition of spacer oil in a geometry shown in Fig. S2B. The same oil as the continuous phase (mineral oil in our case) so it has no effect on the already formed droplets. Changes to the pressure applied to this channel do however impact the overall resistance in the device with in turn impacts the size and rate of droplets produced upstream, so some fine-tuning is required to achieve the desired profile.

Extended length for Droplet analysis and some mixing

This region is mainly to extend the length along which the stable droplets travel. Our devices enables some enhanced droplet mixing by including sharp loops and turns in the channel so the droplets will change direction quickly causing mixing of their contents. Fig. S2C shows an example of such a channel where a droplet moving in one direction abruptly makes a 180 degree turn causing the momentum of its contents to induce mixing.

Droplet collection chamber

Here, we describe the on-chip collection chamber used for storing individual droplets post-analysis. Within the chamber, droplets can self-assemble into a regular lattice, advantageous for high-throughput optical analysis. One collection chamber geometry is shown in Fig. S2D, which includes an optional barrier structure to further limit flow and collect a higher density of droplets.

The main advantage of the collection chamber is enabling delayed analysis of stored particles, achievable through wide-field microscopy or ensemble methods like NMR. Each droplet, containing NDs, remains a separate, confined environment. Fig. S3 provides further detailed images of the collection chamber in brightfield (top) and fluorescence (bottom) under varying optical magnification. Diamond-filled droplets assemble in such a way to be readily analyzed in a wide-field configuration.

C. Microfluidic valve and automation of sample loading

In order to deliver samples to our microfluidic devices consistently and with high repeatability, we use either a custom LabView program or, most commonly, a graphical user interface designed by Fluigent inc. (Oxygen) to control timing, triggering, and pump applied pressures. We use a 10-position fluidic valve (m-switch, Fluigent Inc.) as well as a 10-port manifold which delivers equal pressure to the ten channels of the switch from a single fluidic pressure controller (Flow-EZ, Fluigent). To load a set of samples, we fill centrifuge tubes with the aqueous phase, generally diamond and analyte, and attach them to varying ports on the fluidic valve. Two positions on the switch are reserved for deionized water and for a fluorinated oil in order to form a plug to separate subsequent samples. By pulling oil-water-oil-analyte repeatedly over each sample we effectively “queue up” a run of samples which will arrive at the microfluidic device for analysis.

In our experiment, each sample requires some settling time for the droplet rate to equilibrate after the disturbance of phases changes and momentary pressure interruptions. This delay is approximately 10 min in our experiment and could be shortened with fluidic optimization.

Fig. S4 shows a typical example of loading three individual ND samples onto the chip. Each sample is introduced for 150 s, separated by 100 s intervals of water droplets. The figure presents the raw data from these experiments, predominantly consisting of fluorescence from ND particles. During water droplet intervals, the signal detected is at background levels.

The data display brief changes in FL associated with the introduction of new ND sets onto the chip, resolving in under 5 s due to initial particle loading instability. The FL then stabilizes, maintaining a steady state for extended periods, up to 9 h as necessary. When the M-switch is closed, it induces a sudden pressure change, causing a ripple in the ND flow and associated FL, approximately 10 s before the ND flow ceases and transitions to water droplets.

S3. CONTROL OF DROPLET MODULATION PROFILE

Here we provide further details on controlling droplet modulation profiles in our experiments. The droplet chips feature the ability to control flow profiles by adjusting pressures in various

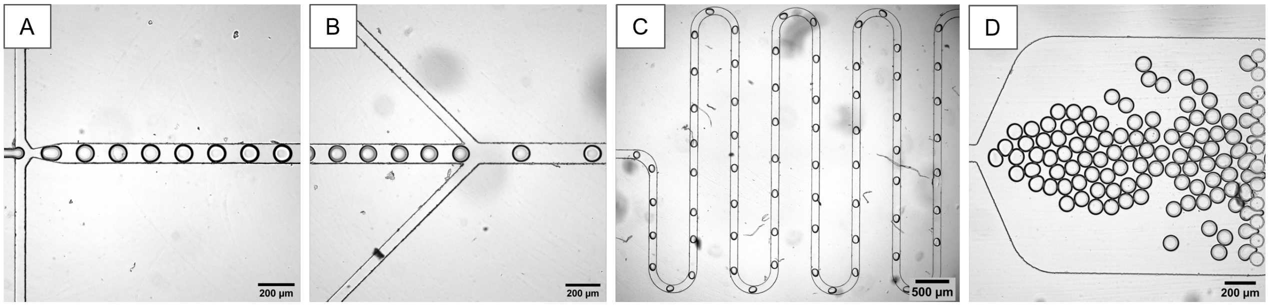

Fig. S2: **Detail of microfluidic devices** used for double lock-in measurements. Our devices include a fluid focusing junction (a) to generate aqueous droplets in an oil continuous phase and a down-chip second oil addition (b) which enables control over the spacing of droplets. As needed, droplet contents can be allowed to mix by following the hairpin turns shown is c, or can be stored by filling the chamber shown in d.

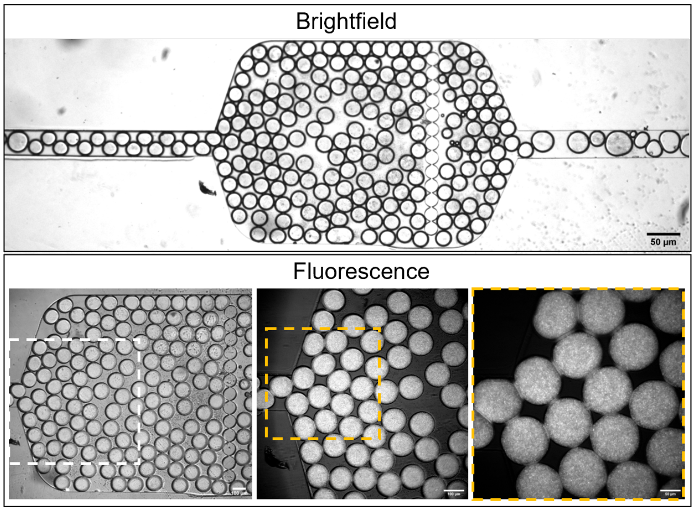

Fig. S3: **Droplet collection chamber.** Panels show droplet collection chamber in brightfiled (top) and fluorescence (bottom) at 10x, 20x and 40x magnification (left to right). Fluorescence from 100 nm NDs is visible using mcherry filter set (ex/em:≈ $540/600nm$).

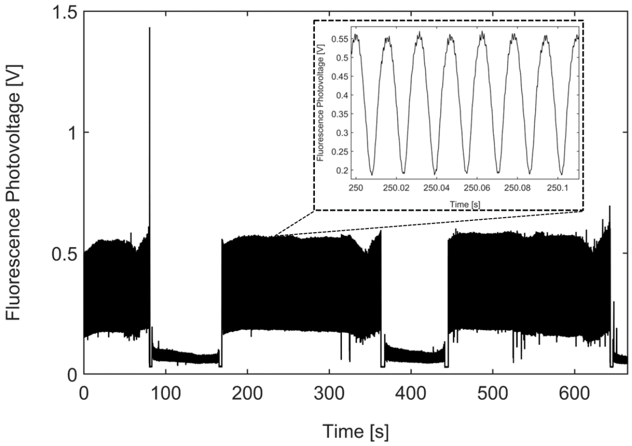

Fig. S4: **Automated sample loading** Three successive ND samples loaded onto the chip. Each sample is loaded for 200 s, punctuated by 75 s of pure water droplets, as visible in raw fluorescence traces. A 10-way valve controls introduction of diamond sample, water, or small spacer plugs of oil to maintain sample separation. Switch operation induces transient ND concentration/fluorescence fluctuations due to line pressure changes, lasting about 2s (opening) and 45s (closing). However, a steady-state region is marked by stable fluorescence, and is maintainable for several hours (Fig. 4). Inset shows the droplet profile over a small time window.

channels. This control is primarily achieved through two methods: injecting spacer oil from junction J_2 (see Fig. 1 of main paper and Fig. S2) and varying the optical spot size for analysis. By altering the amount of spacer oil injected from junction J_2, the shape of the droplet modulation profile can be varied. Additionally, the optical spot size employed constitutes another layer of control. It can be adjusted to be smaller than the droplet diameter, resulting in regions without fluorescence (FL) when the spacer oil passes through the analysis region, or it can be enlarged to cover an area larger than the droplet diameter.

Fig. S6 illustrates two examples of droplet modulation control, along with their Fourier Transform (FT) profiles. In Fig. S6A, the droplet modulation appears nearly sinusoidal, resulting in dominant peaks at f_D in the FT intensity profile (Fig. S6B). The second example (Fig. S6C) shows square-like modulation, producing odd harmonics in the f_D profile (Fig. S6D).

The normalization procedure in Eq. 2 can incorporate the additional f_D harmonics when necessary. The presence of harmonics may assist in analysis, as they help filter out external noise that does not align with the harmonic pattern, thereby improving result accuracy.

S4. IMAGING AND SENSING INSTRUMENTATION

A. Microscope and optics

Fluorescence images and ODMR measurements were performed on a custom-built microscope system that has been reported previously and is briefly described here (*78*). A schematic of the setup is shown in Fig. S7. Around an enclosure and stage (MadCityLabs RM21 Versa), excitation comes from either a 532 nm laser (Coherent Verdi G15) for fluorescence or a goose-neck lamp above the sample for brightfield images. The laser source is reflected by a dichroic mirror (Thorlabs DMLP605R) before being brought to a focus at the back plane of a 20x objective (Olympus LMPLFLN 20x) using a 150 mm focal length lens. The objective impinges the excitation onto a sample held in place on a piezo-position stage (MadCityLabs RM21). Red emission from samples passes through the dichroic and is directed through color filters (600 nm long pass (Thorlabs FEL0600-1), 680/25 nm band pass (Semrock FF01-680/42-25), 735/25

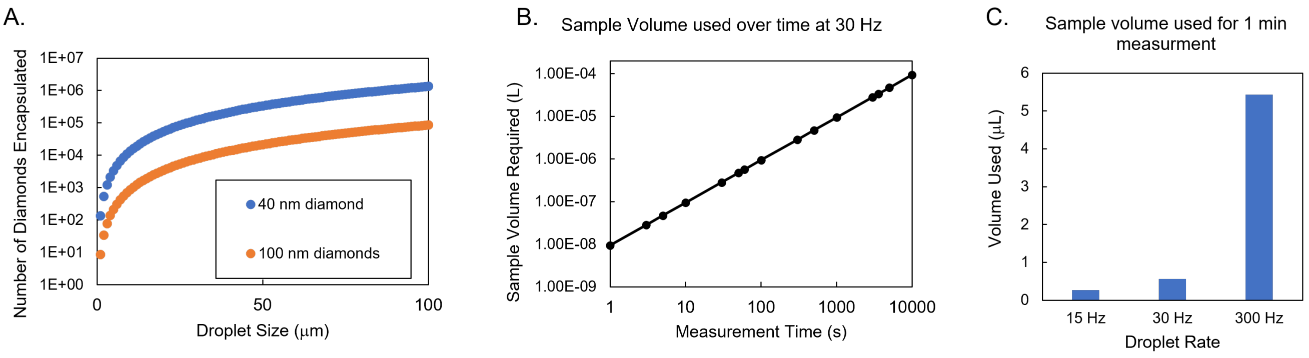

Fig. S5: **Microdroplet and diamond loading parameters.** (A) Calculation reflecting the number of diamond particles encapsulated within a single microdroplet for a given microdroplet size. Values are based on initial concentration of diamond of 0.5 mg/mL for either 40 nm (blue) or 100 nm (orange) diameter diamond particles. (B) Calculated sample volume require to perform double lock-in experiments with droplet rate of 30 Hz for a given measurement time. (C) Calculated sample volume required to perform double lock-in measurements for 1 minute at a given droplet rate

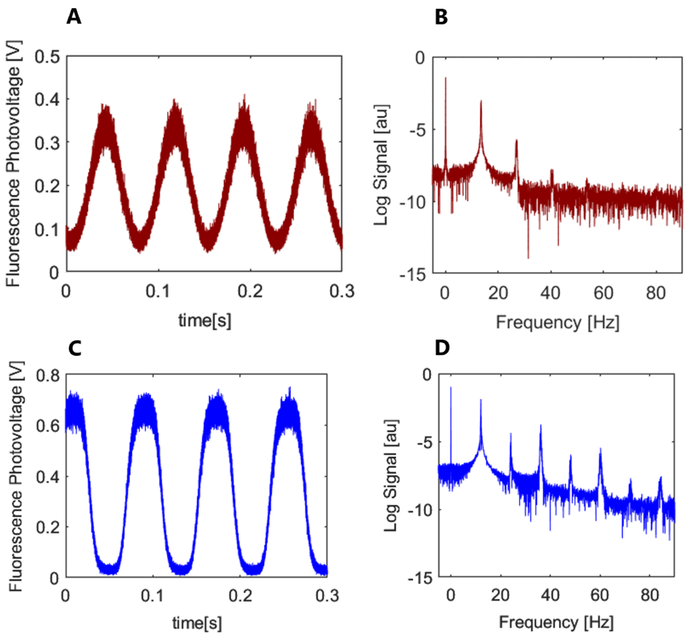

Fig. S6: **Control of droplet flow modulation profiles** achieved by varying the flow rates at the two junctions, J_1 and J_2, and adjusting the optical spot size. A Panel illustrates sinusoidal-like profile. B panel: Fourier intensity spectrum, indicating a single dominant peak at f_D=13.5Hz with lesser peaks at the first hand second harmonics. C Panel displays a more square-like profile, with the FT (D) revealing several harmonics of f_D, predominantly the odd harmonics.

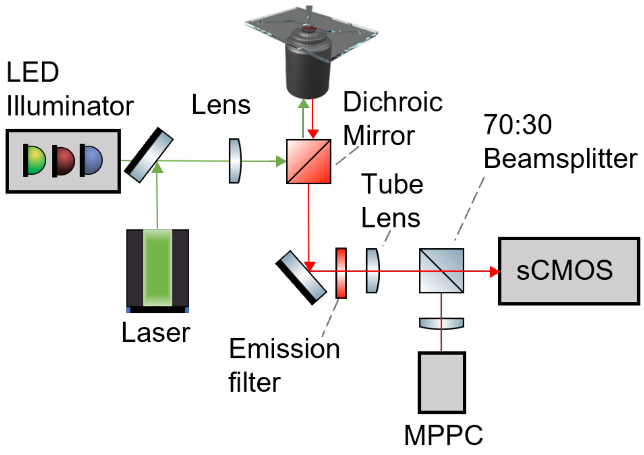

Fig. S7: **Block Diagram** of custom microscope system used for imaging and sensing measurements. Details are in Sec. S4 A

BP (Semrock FF01-735/28) or a combination), before passing through a 200 mm focal length tube lens. A 30:70 beam splitter projects the resulting image onto both an SCMOS camera (Teledyne Kinetix) and toward a focusing lens to a multi-pixel photon counter (Hamamatsu C14452) for detection.

S5. TARGETING NANODIAMOND TO YEAST CELLS

As a demonstration of the potential future impacts of our measurement platform we show that diamond particles can be targeted directly to yeast cells. While effective diamond internalization requires removal of the yeast cell wall, with appropriate functionalization, ND particles can be targeted to the exterior of the yeast via the cell wall. Fig. S8 shows images of the result of such targeting. Here, NDs are coated with Concanavalin A, a protein that interacts with sugar groups on the cell surface. The brightfield image shows the location of 5 *Rhodosporidium toruloides* cells that have been incubated in a solution of Con-A coated NDs. The fluorescence image shows ND emission localized to the cells indicating successful targeting.

S6. BACKGROUND FLUORESCENCE CHARACTERIZATION

The ability to reject background signals is important for increasing the sensitivity of quantum sensing measurements. The measurement of NV center fluorescence changes in the presence of paramagnetic species is a ratiometric measurement in which the drop in fluorescence, measured when MW excitation is on resonance with NV spin transitions, is normalized to total fluoresce emitted from the NV centers. As this total fluorescence value is affected both by signal (NV fluorescence) and by back-

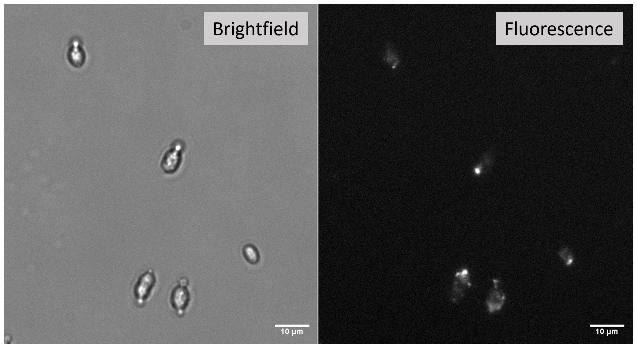

Fig. S8: **Targeting diamond to yeast cells** brightfield (left) and fluorescence (right) images of 100 nm NDs targeted to *R. toruloides* using Concanavalin A. Images collected using CYT5-HQY filter set with emission centered near the NV center maximum at 700 nm.

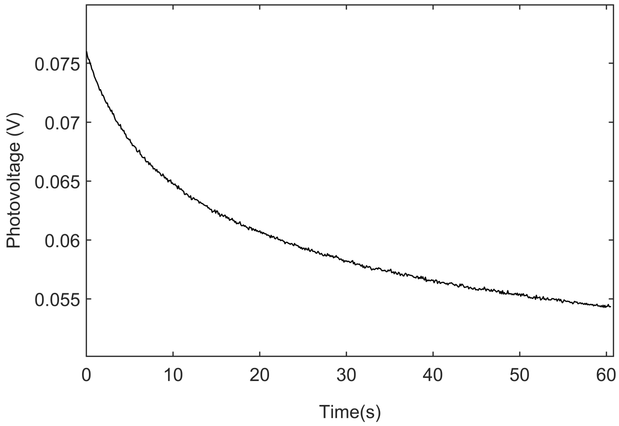

Fig. S9: **Autofluorescence** from a ~100μm region of the PDMS chip without nanodiamond particles measured over 1 minute. Change in autofluorescence over time is at the level of ≈1% of typical ND fluorescence from within the droplets. Suppressing this autofluorescence is crucial for high-precision ODMR measurements.

ground (e.g. scattering, autofluorescence), we characterized the signal from a new PDMS microfluidic device in the absence of NV diamond.

The trace in Fig. S9 shows detected photovoltage from PDMS autofluorescence measured using the same detector and under comparable conditions as the data in the main text. The signal begins at approximately 75 mV and decays over the course of the 1 min measurement window to approximately 55mV. Compared to the photovoltages typically measured from passing droplets (1.2 V for 100 nm NDs), this represents a background signal that can be as much as 6 % of the total and, importantly, can change by as much as 1-2 % over the measurement time. While these exact values will depend on many experimental factors, we note that given that the ODMR percent changes are on the order of 1-5 % fluctuating backgrounds can represent an impediment to improving sensitivity. These data demonstrate the need for excluding static and varying backgrounds that are not at the droplet frequency using our double lock-in method.

S7. PARTICLE TRACKING WITHIN DROPLETS

In this section, the tracking of Brownian motion of 100 nm ND particles within a single droplet, as shown in Fig. 1, is described. A movie capturing a droplet with a sparse distribution of ND particles was analyzed using the trackMate plugin for FIJI software (*40*,*41*). Object size was chosen to be 10 pixels, larger than the actual diameter but allowing some flexability in sizes and objects drift in and out of focus. We then excluded objects below an empirically determined quality threshold. We chose to present trajectories that were monitored for the longest portion of the movie without being interrupted in an attempt to avoid biasing short trajectories. The analysis, spanning a 30 s period, focuses on the motion of individual particles. It is assumed that the particles are dispersed enough to prevent much overlap in their trajectories of motion, assessed by frame-to-frame movement comparisons within the movie.

The results, depicted in Fig. 1E, reveal trajectories of the particles spanning ~5 μm, far larger than the diameter of the ND particles. This analysis, when extended to a larger set of droplets, facilitates the creation of a histogram of droplet motion, shown in Fig. 1Eii. We note that the tracking is limited to two dimensions. Movements in the third dimension cause blurring of the particles as they exit the imaging focal plane, and occasionally results in losing a track. However, the focal region is wide enough to capture most trajectories during the 30s interval.

S8. VIDEOS OF FLOWING DROPLETS

Fig. S10 presents videos illustrating the motion of 3 μm particles (Fig. S10A) and 100 nm ND-filled droplets flowing at various controlled speeds (Fig. S10B-D).

Fig. S10 shows still frame grabs from these videos, while the videos themselves are available in Supplementary Movie 5. In Fig. S10B-D, a small amount of white light is introduced into the imaging system to outline the droplets, and the imaging optical spot is set to be slightly smaller than the droplet diameter. The bright signal then observed is almost exclusively from the ND fluorescence.

Fig. S10A demonstrates the uniform distribution of NDs within a droplet, which can be seen moving and sampling the droplet volume in Supplementary Movie 5. Fig. S10B-D feature movies showing droplets at varying flow rates: slow, medium, and fast. The droplets form a regular array-like pattern, and stroboscopic sampling gives the impression of each droplet sequentially replacing the next. In Fig. S10B, we form a single stream of droplets, comparable to the experiments in the main text. To achieve faster flow rates we use a device that generates an array of droplets 2-wide that is able to achieve higher flow rates (Fig. S10C is measured at the same rate as Fig. S10A, because our spot size only samples one row of droplets). In faster flows (Fig. S10C-D), the droplet movement outpaces the camera's exposure time, causing blurred images. Importantly, the measured signal from all three of these videos yields a stable oscillating signal (main text) highlighting the precision and versatility of droplet-based approaches, particularly in consistently loading droplets with ND particles.

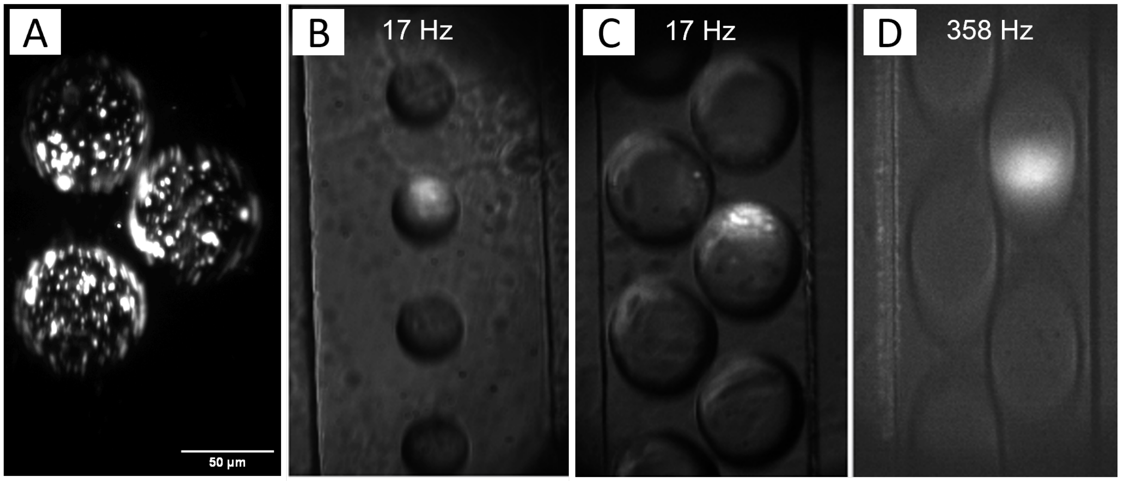

Fig. S10: **Movies of droplet flow with NDs.** Still frame grabs are shown, full movies are in Supplementary Movie 5, featuring (A) Fluorescence video of flowing droplets with 750 nm ND particles. (B-D) Fluorescence videos of 100 nm ND particles. Small amount of white light was introduced to make clear the droplet boundaries. Flow is highly regular and orderly. (C-D) At higher speeds, the droplets self-assemble into a double rail configuration, as seen in the last panel. For (D), flow rate exceeds the camera dwell time, resulting in images that appear blurred.

S9. PARTICLE NUMBER VARIATION PER DROPLET

By analyzing fluorescence modulation from flowing droplets, we approximate the number variation of ND particles per droplet. The method involves collecting isolating the 1 KHz band of the Fourier spectrum in Fig. 4, which contains information about the number of NDs in the field of view at any given time. By plotting a histogram of the variations in intensity of the Fourier band, we estimate an upper bound for the percentage variation in ND loading over time. The data shown in the histogram in Fig. 4D represent this variability in the Fourier spectrum generated using time bins corresponding to ~ 200 droplets. However, longer time bins will reduce the calculated σ by $1/\sqrt{t}$ until the value is no longer limited by the time-bin size. A reasonable estimate of the maximum time bin is given by the Allan deviation in Fig. 4C. Based on this approach, we calculate the variation between droplets to be 0.23%. This variation corresponds to an estimated 2,300 particles per droplet. Despite the potential overestimation, this finding underscores the high degree of control attainable in ND loading within the droplets, highlighting the precision of our experimental setup.

S10. ALLAN VARIANCE ANALYSIS

We describe the details of Allan variance analysis of the time-dependent droplet PL time series. The PL time series (see main text, Eq. 1) for the flowing droplets has been divided into smaller time bins of size Δt for observing variations in amplitude of the Fourier spectrum in the relevant frequency bands. This time-averaged normalized contrast has been evaluated as

$$C(t_i) = \frac{\mathcal{F}(f_{\mathrm{MW}} \pm f_D, t_i)}{2\mathcal{F}(f_D, t_i)} \qquad (3)$$

where $\mathcal{F}(f, \Delta t_i)$ term describes the amplitude of Fourier frequency f in time between t_i and $t - \Delta t_i$, extracted through the *fft* numerical algorithm in MATLAB. The DC component of the PL time trace in each time bin Δt has been subtracted and therefore, contribution of minor fluctuations from droplet rate is minimized in the contrast calculations. Allan variance is evaluated from the above $C(t_i)$ time-series metric. In context of clock stability literature (*80*), we assume our ODMR contrast timepoints to be fractional frequency deviations. The governing exponent β of the Allan variance $\sigma(\tau)$ is related to the Fourier frequency exponent α as $\beta = \alpha - 1$. For uncorrelated white Gaussian time-domain noise, the logarithmic scale Allan deviation curve shows $-\frac{1}{2}$ linear slope. Our droplet flow contrast time series attains stable Gaussian noise scaling for several minutes, as shown in Fig. 4C. A MATLAB repository code *allan* (*81*) was used to evaluate Allan deviations.

S11. COMPARISON OF RELATED METHODS OF PARAMAGNETIC ION SENSING

We note before expanding in detail that the methods compiled here are not exhaustive of the literature, but rather those that are the highest performing or most relevant comparisons to the goals of our work. (*17*,*20*,*44*,*58*,*61*,*82–84*) It should be noted that while many groups aim to detect paramagnetic species, the methods used, the conditions of the experiments, and often the values and units chosen to report are highly variable. As such, a direct comparison between measurements can be dubious, especially across different physical substrates/sensing platforms and across different optical experiment conditions. Indeed, while some groups aim to accelerate measurement times, others aim for superlative sensitivity, while still others aim to decrease sample volume required. As a result, one reference (e.g. Ref. (*84*)) may report a sensitivity of 1 μM in the absence of attempts to measure lower while others (e.g. Ref. (*58*)) report high sensitivity while not indicating an intention to miniaturize.

With those caveats in place, we highlight a few relevant works in Table S1, and compare results on axes of parameters that we feel are important to the measurement. We make note that Ref. (*17*) report very high sensitivity to gadolinium ions; however, they also include a thorough (and quite helpful) discussion of the impact of adsorption on the diamond surface. We be-

Reference	*Substrate*	*Method*	*Analyte*	*Lowest [] Measured**	*Meaurement Time*	*Sample Volume***	
This Work	**40 nm NVND**	**In-flow ODMR**	**$GdCl_3$**	**500 nM**	**2 min**	**540 nL**	**NVND**
(58)	70 nm NVND	T1	$GdCl_3$	1 nM Gd	8 min	200 uL	
(82)	50 nm NVND	T1 / single Tau T1	Gd^{3+} (as $Gd(ClO_4)_3$)	1 mM	10s	NR	
(83)	30 nm NVND, cleavable polymer coatings	T1	Gd3+ as cleavable coating	1 mM (theory)	Approx. 2 min	NR	
(84)	70 nm NVND	T1	TEMPO / Nitroxyl	10-90 spins per particle	1-2 min	7 uL	
(44)	Diamond chip - 15 nm implantation depth	PL Contrast MW on/off	$GdCl_3$	1 uM GdCl	8.7 s	100uL	**Single crystal Diamond chip**
(61)	Diamond chip- 6 nm implantation depth	Single Tau T1	Gd^{3+}	80 uM	20s	20 uL	
(20)	Diamond chip - 5 nm NV layer	T1	Gd^{3+}	1 uM	Approx 1 min	600nL	
(17)	Microfluidic device on Diamond chip	Single Tau T1	Mn^{2+} as $MnCl_2$	20 nM	5s	25 uL	

* This table reports the lowest concentration reported in data from these references, not any calculated, theoretical, or extrapolated LOD

** Sample volumes in the table refer to the actual volume used in each measurement, not any theoretical minimum detection volume; NR indicates that volume was not reported

Supplementary Table S1: Comparison of some related works from literature. Table is organized separating nanodiamond-based methods (grey boxes) and single crystal diamond-based methods (white boxes). T_1- based measurements (method column) collect a full T_1 curve and fit to determine T_1 relaxation times while single Tau T_1 methods monitor PL changes at a single point along the T_1 relaxation curve. Analyte column refers to the analyte measured in "Lowest [] measured" column.

lieve that this complicates the exact value reported, in that while it is certainly true that gadolinium is detected from the 20 nM solution in flow, whether the steady state of that signal is distinguishable from a 100 nM or 500 nM solution is not immediately apparent. We suspect that this effect is at play to some extent on many of these measurements in this table, especially those that use negatively charged diamond to detect paramagnetic cations (e.g. Ref. (*58*)). This complicating factor is one reason we approached TEMPOL measurements, which do not have the same adsorption interactions at the diamond surface.

This table highlights the unique benefits of our measurement geometry. Our microfluidic platform enables the combination of high sensitivity, small sample sizes/volumes and short measurement time. While the methods listed each have their own benefits and merit for a variety of purposes, the aspects of our platform that we highlight make it a powerful measurement tool for high throughput and high sensitivity analyte detection.

S12. SUPPLEMENTAL MOVIES

Supplemental Movie 1 Movie shows the trajectories of selected 100 nm fluorescent diamond particles as they move within a stationary microdroplet. The droplet (water droplet in oil) is approximately 100 um in diameter and the particles traverse an average of approximately 5 um over 30 s. Tracking performed using TrackMate. (*40*,*41*)

Supplemental Movie 2 Brightfield movies of microdroplets flowing through the different structures on our device including 1: droplet generation junction, 2: droplet spacing junction, 3: Droplet mixing loops, 4: droplet storage chamber. Framerate is slowed for clarity.

Supplemental Movie 3 Fluorescence movies of microdroplets flowing through the microfluidic structures also shown in Supplemental movie 2 including 1: droplet generation junction, 2: droplet spacing junction, 3: Droplet mixing loops, 4: droplet storage chamber. Framerate is slowed for clarity.

Supplemental Movie 4 Fluorescence Movie of 750 nm diamond loaded into flowing droplets approximately 50 μm in diameter.

Supplemental Movie 5 Fluorescence Videos corresponding to stills shown in supplementary figure Fig. S10 including 4 parts - 1: Droplets containing 750 nm sized diamond in flowing droplets. 2-4: fluorescence videos of diamond-filled droplets flowing at varying rates and droplet profiles. The last video showing 350 Hz droplets is blurred from droplets moving faster than the camera framerate.

Supplemental Movie 6 Brightfield movie of yeast cells encapsulated in flowing microdroplets. Droplets and cells are both transparent, but cells and clusters of cells can be seen as small objects inside each droplet.

REFERENCES AND NOTES

1. C. L. Degen, F. Reinhard, P. Cappellaro, Quantum sensing. *Rev. Mod. Phys.* **89**, 035002 (2017).

2. T. Zhang, G. Pramanik, K. Zhang, M. Gulka, L. Wang, J. Jing, F. Xu, Z. Li, Q. Wei, P. Cigler, Z. Chu, Toward quantitative biosensing with nitrogen–vacancy center in diamond. *ACS Sens.* **6**, 2077–2107 (2021).

3. M. W. Doherty, F. Dolde, H. Fedder, F. Jelezko, J. Wrachtrup, N. B. Manson, L. C. L. Hollenberg, Theory of the ground-state spin of the NV center in diamond. *Phys. Rev. B* **85**, 205203 (2012).

4. F. Jelezko, J. Wrachtrup, Single defect centres in diamond: A review. *Phys. Status Solidi A Appl. Mater. Sci.* **203**, 3207–3225 (2006).

5. P. Neumann, I. Jakobi, F. Dolde, C. Burk, R. Reuter, G. Waldherr, J. Honert, T. Wolf, A. Brunner, J. H. Shim, D. Suter, H. Sumiya, J. Isoya, J. Wrachtrup, High-precision nanoscale temperature sensing using single defects in diamond. *Nano Lett.* **13**, 2738–2742 (2013).

6. G. Kucsko, P. Maurer, N. Y. Yao, M. Kubo, H. Noh, P. Lo, H. Park, M. D. Lukin, Nanometre-scale thermometry in a living cell. *Nature* **500**, 54–58 (2013).

7. J. Choi, H. Zhou, R. Landig, H.-Y. Wu, X. Yu, S. E. Von Stetina, G. Kucsko, S. E. Mango, D. J. Needleman, A. D. Samuel, P. C. Maurer, H. Park, M. D. Lukin, Probing and manipulating embryogenesis via nanoscale thermometry and temperature control. *Proc. Natl. Acad. Sci. U.S.A.* **117**, 14636–14641 (2020).

8. G. Petrini, E. Moreva, E. Bernardi, P. Traina, G. Tomagra, V. Carabelli, I. P. Degiovanni, M. Genovese, Is a quantum biosensing revolution approaching? Perspectives in NV-assisted current and thermal biosensing in living cells. *Adv. Quantum Technol.* **3**, 2000066 (2020).

9. K. J. van der Laan, A. Morita, F. P. Perona-Martinez, R. Schirhagl, Evaluation of the oxidative stress response of aging yeast cells in response to internalization of fluorescent nanodiamond biosensors. *Nanomaterials* **10**, 372 (2020).

10. R. Sharmin, T. Hamoh, A. Sigaeva, A. Mzyk, V. G. Damle, A. Morita, T. Vedelaar, R. Schirhagl, Fluorescent nanodiamonds for detecting free-radical generation in real time during shear stress in human umbilical vein endothelial cells. *ACS Sens.* **6**, 4349–4359 (2021).

11. B. S. Miller, L. Bezinge, H. D. Gliddon, D. Huang, G. Dold, E. R. Gray, J. Heaney, P. J. Dobson, E. Nastouli, J. J. Morton, R. A. McKendry, Spin-enhanced nanodiamond biosensing for ultrasensitive diagnostics. *Nature* **587**, 588–593 (2020).

12. D. R. Glenn, D. B. Bucher, J. Lee, M. D. Lukin, H. Park, R. L. Walsworth, High-resolution magnetic resonance spectroscopy using a solid-state spin sensor. *Nature* **555**, 351–354 (2018).

13. N. Aslam, M. Pfender, P. Neumann, R. Reuter, A. Zappe, F. F. de Oliveira, A. Denisenko, H. Sumiya, S. Onoda, J. Isoya, J. Wrachtrup, Nanoscale nuclear magnetic resonance with chemical resolution. *Science* **357**, 67–71 (2017).

14. T. Staudacher, F. Shi, S. Pezzagna, J. Meijer, J. Du, C. A. Meriles, F. Reinhard, J. Wrachtrup, Nuclear magnetic resonance spectroscopy on a (5-nanometer)3 sample volume. *Science* **339**, 561–563 (2013).

15. H. J. Mamin, M. Kim, M. H. Sherwood, C. T. Rettner, K. Ohno, D. D. Awschalom, D. Rugar, Nanoscale nuclear magnetic resonance with a nitrogen-vacancy spin sensor. *Science* **339**, 557–560 (2013).

16. J. Tetienne, L. Rondin, P. Spinicelli, M. Chipaux, T. Debuisschert, J. Roch, V. Jacques, Magnetic-field-dependent photodynamics of single NV defects in diamond: An application to qualitative alloptical magnetic imaging. *New J. Phys.* **14**, 103033 (2012).

17. F. C. Ziem, N. S. Gotz, A. Zappe, S. Steinert, J. Wrachtrup, Highly sensitive detection of physiological spins in a microfluidic device. *Nano Lett.* **13**, 4093–4098 (2013).

18. J. Smits, J. T. Damron, P. Kehayias, A. F. McDowell, N. Mosavian, I. Fescenko, N. Ristoff, A. Laraoui, A. Jarmola, V. M. Acosta, Two-dimensional nuclear magnetic resonance spectroscopy with a microfluidic diamond quantum sensor. *Sci. Adv.* **5**, eaaw7895 (2019).

19. V. Bharadwaj, O. Jedrkiewicz, J. Hadden, B. Sotillo, M. R. Vazquez, P. Dentella, T. T. Fernandez, A. Chiappini, A. N. Giakoumaki, T. Le Phu, M. Bollani, M. Ferrari, R. Ramponi, P. E. Barclay, S. M. Eaton, Femtosecond laser written photonic and microfluidic circuits in diamond. *JPhys. Photonics* **1**, 022001 (2019).

20. R. D. Allert, F. Bruckmaier, N. R. Neuling, F. A. Freire-Moschovitis, K. S. Liu, C. Schrepel, P. Schätzle, P. Knittel, M. Hermans, D. B. Bucher, Microfluidic quantum sensing platform for lab-on-a-chip applications. *Lab Chip* **22**, 4831–4840 (2022).

21. A. M. Schrand, H. Huang, C. Carlson, J. J. Schlager, E. Ōsawa, S. M. Hussain, L. Dai, Are diamond nanoparticles cytotoxic? *J. Phys. Chem. B* **111**, 2–7 (2007).

22. O. A. Shenderova, A. I. Shames, N. A. Nunn, M. D. Torelli, I. Vlasov, A. Zaitsev, Synthesis, properties, and applications of fluorescent diamond particles. *J. Vac. Sci. Technol. B, Nanotechnol. Microelectron.* **37**, 030802 (2019).

23. I. P. Chang, K. C. Hwang, J. A. Ho, C.-C. Lin, R. J.-R. Hwu, J.-C. Horng, Facile surface functionalization of nanodiamonds. *Langmuir* **26**, 3685–3689 (2010).

24. K.-K. Liu, C.-L. Cheng, C.-C. Chang, J.-I. Chao, Biocompatible and detectable carboxylated nanodiamond on human cell. *Nanotechnology* **18**, 325102 (2007).

25. T. Zhang, A. Neumann, J. Lindlau, Y. Wu, G. Pramanik, B. Naydenov, F. Jelezko, F. Schuder, S. Huber, M. Huber, F. Stehr, A. Högele, T. Weil, T. Liedl, Dna-based self-assembly of fluorescent nanodiamonds. *J. Am. Chem. Soc.* **137**, 9776–9779 (2015).

26. G. Reina, L. Zhao, A. Bianco, N. Komatsu, Chemical functionalization of nanodiamonds: Opportunities and challenges ahead. *Angew. Chem. Intl. Ed. Engl.* **58**, 17918–17929 (2019).

27. Y. Zhang, A. A. Tamijani, M. E. Taylor, B. Zhi, C. L. Haynes, S. E. Mason, R. J. Hamers, Molecular surface functionalization of carbon materials via radical-induced grafting of terminal alkenes. *J. Am. Chem. Soc.* **141**, 8277–8288 (2019).

28. Z. R. Jones, N. J. Niemuth, Y. Zhang, C. R. Protter, P. C. Kinsley, R. D. Klaper, R. J. Hamers, Use of magnetic modulation of nitrogen-vacancy center fluorescence in nanodiamonds for quantitative analysis of nanoparticles in organisms. *ACS Meas. Sci. Au* **2**, 351–360 (2022).

29. Q. Zheng, X. Shi, J. Jiang, H. Mao, N. Montes, N. Kateris, J. A. Reimer, H. Wang, H. Zheng, Unveiling the complexity of nanodiamond structures. *Proc. Natl. Acad. Sci. U.S.A.* **120**, e2301981120 (2023).

30. S. Chowdhury, M. Chen, R. Eckert, D. Ren, F. Wu, N. Repina, L. Waller, High-resolution 3D refractive index microscopy of multiple-scattering samples from intensity images. *Optica* **6**, 1211–1219 (2019).

31. L. V. H. Rodgers, L. B. Hughes, M. Xie, P. C. Maurer, S. Kolkowitz, A. C. Bleszynski Jayich, N. P. de Leon, Materials challenges for quantum technologies based on color centers in diamond. *MRS Bulletin* **46**, 623–633 (2021).

32. A. Huebner, S. Sharma, M. Srisa-Art, F. Hollfelder, J. B. Edel, A. J. Demello, Microdroplets: A sea of applications? *Lab Chip* **8**, 1244 (2008).

33. A. B. Theberge, F. Courtois, Y. Schaerli, M. Fischlechner, C. Abell, F. Hollfelder, W. T. Huck, Microdroplets in microfluidics: An evolving platform for discoveries in chemistry and biology. *Angew. Chem. Int. Ed. Engl.* **49**, 5846–5868 (2010).

34. K. Fang, V. M. Acosta, C. Santori, Z. Huang, K. M. Itoh, H. Watanabe, S. Shikata, R. G. Beausoleil, High-sensitivity magnetometry based on quantum beats in diamond nitrogen-vacancy centers. *Phys. Rev. Lett.* **110**, 130802 (2013).

35. T. Wolf, P. Neumann, K. Nakamura, H. Sumiya, T. Ohshima, J. Isoya, J. Wrachtrup, Subpicotesla diamond magnetometry. *Phys. Rev. X* **5**, 041001 (2015).

36. D. T. Chiu, R. M. Lorenz, Chemistry and biology in femtoliter and picoliter volume droplets. *Acc. Chem. Res.* **42**, 649–658 (2009).

37. R. O. Grigoriev, M. F. Schatz, V. Sharma, Chaotic mixing in microdroplets. *Lab Chip* **6**, 1369–1372 (2006).

38. M. Srisa-Art, A. J. DeMello, J. B. Edel, Fluorescence lifetime imaging of mixing dynamics in continuous-flow microdroplet reactors. *Phys. Rev. Lett.* **101**, 014502 (2008).

39. M. F. Shlesinger, B. West, J. Klafter, Levy dynamics of enhanced diffusion: Application to turbulence. *Phys. Rev. Lett.* **58**, 1100–1103 (1987).

40. S. R. Hemelaar, A. Nagl, F. Bigot, M. M. Rodriguez-Garcia, M. P. de Vries, M. Chipaux, R. Schirhagl, The interaction of fluorescent nanodiamond probes with cellular media. *Microchim. Acta* **184**, 1001–1009 (2017).

41. V. Radu, J. C. Price, S. J. Levett, K. K. Narayanasamy, T. D. Bateman-Price, P. B. Wilson, M. L. Mather, Dynamic quantum sensing of paramagnetic species using nitrogen-vacancy centers in diamond. *ACS Sens.* **5**, 703–710 (2020).

42. J. Goree, Double lock-in detection for recovering weak coherent radio frequency signals. *Rev. Sci. Instrum.* **56**, 1662–1664 (1985).

43. M. Fujiwara, A. Dohms, K. Suto, Y. Nishimura, K. Oshimi, Y. Teki, K. Cai, O. Benson, Y. Shikano, Real-time estimation of the optically detected magnetic resonance shift in diamond quantum thermometry toward biological applications. *Phys. Rev. Res.* **2**, 043415 (2020).

44. C. A. Hart, J. M. Schloss, M. J. Turner, P. J. Scheidegger, E. Bauch, R. L. Walsworth, N-□–diamond magnetic microscopy using a double quantum 4-ramsey protocol. *Phys. Rev. Appl.* **15**, 044020 (2021).

45. Q. Gu, L. Shanahan, J. W. Hart, S. Belser, N. Shofer, M. Ataure, H. S. Knowles, Simultaneous nanorheometry and nanothermometry using intracellular diamond quantum sensors. *ACS Nano* **17**, 20034–20042 (2023).

46. S. K. Singam, J. Motylewski, A. Monaco, E. Gjorgievska, E. Bourgeois, M. Nesladek, M. Giugliano, E. Goovaerts, Contrast induced by a static magnetic field for improved detection in nanodiamond fluorescence microscopy. *Phys. Rev. Appl.* **6**, 064013 (2016).

47. Z. R. Jones, N. J. Niemuth, M. E. Robinson, O. A. Shenderova, R. D. Klaper, R. J. Hamers, Selective imaging of diamond nanoparticles within complex matrices using magnetically induced fluorescence contrast. *Environ. Sci. Nano* **7**, 525–534 (2020).

48. M. D. Torelli, N. A. Nunn, Z. R. Jones, T. Vedelaar, S. K. Padamati, R. Schirhagl, R. J. Hamers, A. I. Shames, E. O. Danilov, A. Zaitsev, O. A. Shenderova, High temperature treatment of diamond particles toward enhancement of their quantum properties. *Front. Phys.* **8**, 205 (2020).

49. T. Petit, L. Puskar, T. Dolenko, S. Choudhury, E. Ritter, S. Burikov, K. Laptinskiy, Q. Brzustowski, U. Schade, H. Yuzawa, M. Nagasaka, N. Kosugi, M. Kurzyp, A. Venerosy, H. Girard, J. C. Arnault, E. Osawa, N. Nunn, O. Shenderova, E. F. Aziz, Unusual water hydrogen bond network around hydrogenated nanodiamonds. *J. Phys. Chem. C* **121**, 5185–5194 (2017).

50. Y. E. Nesmelov, A. Gopinath, D. D. Thomas, Aqueous sample in an EPR cavity: Sensitivity considerations. *J. Magn. Reson.* **167**, 138–146 (2004).

51. M. M. Roessler, E. Salvadori, Principles and applications of EPR spectroscopy in the chemical sciences. *Chem. Soc. Rev.* **47**, 2534–2553 (2018).

52. N. Abhyankar, A. Agrawal, J. Campbell, T. Maly, P. Shrestha, V. Szalai, Recent advances in microresonators and supporting instrumentation for electron paramagnetic resonance spectroscopy. *Rev. Sci. Instrum.* **93**, 101101 (2022).

53. J. Wertz, J. R. Bolton, Electron Spin Resonance: Elementary Theory and Practical Applications (Springer Dordrecht, 1986).

54. F. Perona Martinez, A. C. Nusantara, M. Chipaux, S. K. Padamati, R. Schirhagl, Nanodiamond relaxometry-based detection of free-radical species when produced in chemical reactions in biologically relevant conditions. *ACS Sens.* **5**, 3862–3869 (2020).

55. A. Sigaeva, H. Shirzad, F. Perona-Martinez, A. Citra Nusantara, N. Mougios, M. Chipaux, R. Schirhagl, Diamond-based nanoscale quantum relaxometry for sensing free radical production in cells. *Small* **18**, 2105750 (2022).

56. J. Strömqvist, A. Chmyrov, S. Johansson, A. Andersson, L. Mäler, J. Widengren, Quenching of triplet state fluorophores for studying diffusion-mediated reactions in lipid membranes. *Biophys. J.* **99**, 3821–3830 (2010).

57. S. Steinert, F. Ziem, L. T. Hall, A. Zappe, M. Schweikert, N. Götz, A. Aird, G. Balasubramanian, L. Hollenberg, J. Wrachtrup, Magnetic spin imaging under ambient conditions with sub-cellular resolution. *Nat. Commun.* **4**, 1607 (2013).

58. E. S. Grant, M. B. A. Olia, Y. Li, E. P. Walsh, G. Mccoll, L. T. Hall, D. A. Simpson, Method for in-solution, highthroughput □1 relaxometry using fluorescent nanodiamonds. *Phys. Rev. Appl.* **20**, 034018 (2023).

59. R. D. Davis, M. I. Jacobs, F. A. Houle, K. R. Wilson, Colliding-droplet microreactor: Rapid on-demand inertial mixing and metalcatalyzed aqueous phase oxidation processes. *Anal. Chem.* **89**, 12494–12501 (2017).

60. J. K. Lee, S. Banerjee, H. G. Nam, R. N. Zare, Acceleration of reaction in charged microdroplets. *Q. Rev. Biophys.* **48**, 437–444 (2015).

61. B. Kintses, C. Hein, M. F. Mohamed, M. Fischlechner, F. Courtois, C. Laine, F. Hollfelder, Picoliter cell lysate assays in microfluidic droplet compartments for directed enzyme evolution. *Chem. Biol.* **19**, 1001–1009 (2012).

62. A. M. Wojciechowski, M. Karadas, A. Huck, C. Osterkamp, S. Jankuhn, J. Meijer, F. Jelezko, U. L. Andersen, Contributed review: Camera-limits for wide-field magnetic resonance imaging with a nitrogen-vacancy spin sensor. *Rev. Sci. Instrum.* **89**, 031501 (2018).

63. M. Parashar, A. Bathla, D. Shishir, A. Gokhale, S. Bandyopadhyay, K. Saha, Sub-second temporal magnetic field microscopy using quantum defects in diamond. *Sci. Rep.* **12**, 8743 (2022).

64. J. L. Webb, L. Troise, N. W. Hansen, L. F. Frellsen, C. Osterkamp, F. Jelezko, S. Jankuhn, J. Meijer, K. Berg-Sørensen, J.-F. Perrier, A. Huck, U. L. Andersen, High-speed wide-field imaging of microcircuitry using nitrogen vacancies in diamond. *Phys. Rev. Appl.* **17**, 064051 (2022).

65. N. Shembekar, C. Chaipan, R. Utharala, C. A. Merten, Droplet-based microfluidics in drug discovery, transcriptomics and high-throughput molecular genetics. *Lab Chip* **16**, 1314–1331 (2016).

66. A. L. Givan, Flow cytometry: An introduction, in *Flow Cytometry Protocols*, Eds. T. S. Hawley and R. G. Hawley (Humana Press, Totowa, NJ, 2011) pp. 1–29.

67. E. Eruslanov, S. Kusmartsev, Identification of ros using oxidized dcfda and flow-cytometry, in *Advanced Protocols in Oxidative Stress II*, Ed. D. Armstrong (Humana Press, Totowa, NJ, 2010) pp. 57–72.

68. J. F. Barry, J. M. Schloss, E. Bauch, M. J. Turner, C. A. Hart, L. M. Pham, R. L. Walsworth, Sensitivity optimization for nvdiamond magnetometry. *Rev. Mod. Phys.* **92**, 015004 (2020).

69. A. Ajoy, P. Cappellaro, Stable three-axis nuclear-spin gyroscope in diamond. *Phys. Rev. A* **86**, 062104 (2012).

70. A. Jarmola, S. Lourette, V. M. Acosta, A. G. Birdwell, P. Blümler, D. Budker, T. Ivanov, V. S. Malinovsky, Demonstration of diamond nuclear spin gyroscope. *Sci. Adv.* **7**, eabl3840 (2021).

71. A. Ajoy, K. Liu, R. Nazaryan, X. Lv, P. R. Zangara, B. Safvati, G. Wang, D. Arnold, G. Li, A. Lin, P. Raghavan, E. Druga, S. Dhomkar, D. Pagliero, J. A. Reimer, D. Suter, C. A. Meriles, A. Pines, Orientation-independent room temperature optical 13c hyperpolarization in powdered diamond. *Sci. Adv.* **4**, eaar5492 (2018).

72. W. Beatrez, O. Janes, A. Akkiraju, A. Pillai, A. Oddo, P. Reshetikhin, E. Druga, M. McAllister, M. Elo, B. Gilbert, D. Suter, A. Ajoy, Floquet prethermalization with lifetime exceeding 90 s in a bulk hyperpolarized solid. *Phys. Rev. Lett.* **127**, 170603 (2021).

73. O. Sahin, E. de Leon Sanchez, S. Conti, A. Akkiraju, P. Reshetikhin, E. Druga, A. Aggarwal, B. Gilbert, S. Bhave, A. Ajoy, High field magnetometry with hyperpolarized nuclear spins. *Nat. Commun.* **13**, 5486 (2022).

74. B. W. Blankenship, Z. R. Jones, N. Zhao, H. Singh, A. Sarkar, R. Li, L. Suh, A. Chen, C. P. Grigoropoulos, A. Ajoy, Complex three-dimensional microscale structures for quantum sensing applications. *Nano Lett.* **23**, 9272–9279 (2023).

75. D. Ershov, M.-S. Phan, J. W. Pylvänäinen, S. U. Rigaud, L. L. Blanc, A. Charles-Orszag, J. R. W. Conway, R. F. Laine, N. H. Roy, D. Bonazzi, G. Duménil, G. Jacquemet, J.-Y. Tinevez, Trackmate 7: Integrating state-of-the-art segmentation algorithms into tracking pipelines. *Nat. Methods* **19**, 829–832 (2022).

76. J.-Y. Tinevez, N. Perry, J. Schindelin, G. M. Hoopes, G. D. Reynolds, E. Laplantine, S. Y. Bednarek, S. L. Shorte, K. W. Eliceiri, Trackmate: An open and extensible platform for singleparticle tracking. *Methods* **115**, 80–90 (2017).

77. A. Kumar, C. K. Dixit, Methods for characterization of nanoparticles, in *Advances in Nanomedicine for the Delivery of Therapeutic Nucleic Acids* (Woodhead Publishing, 2017) pp. 43–58.

78. D. W. Allan, Time and frequency (time-domain) characterization, estimation, and prediction of precision clocks and oscillators. *IEEE Trans. Ultrason. Ferroelectr. Freq. Control* **34**, 647–654 (1987).

79. M. Hopcroft, allan (https://mathworks.com/matlabcentral/fileexchange/13246-allan), MATLAB Central File Exchange, MATLAB Central file exchange (23 February 2024).

80. J.-P. Tetienne, T. Hingant, L. Rondin, A. Cavaillès, L. Mayer, G. Dantelle, T. Gacoin, J. Wrachtrup, J.-F. Roch, V. Jacques, Spin relaxometry of single nitrogen-vacancy defects in diamond nanocrystals for magnetic noise sensing. *Phys. Rev. B* **87**, 235436 (2013).

81. T. Rendler, J. Neburkova, O. Zemek, J. Kotek, A. Zappe, Z. Chu, P. Cigler, J. Wrachtrup, Optical imaging of localized chemical events using programmable diamond quantum nanosensors. *Nat. Commun.* **8**, 14701 (2017).

82. J. Barton, M. Gulka, J. Tarabek, Y. Mindarava, Z. Wang, J. Schimer, H. Raabova, J. Bednar, M. B. Plenio, F. Jelezko, M. Nesladek, P. Cigler, Nanoscale dynamic readout of a chemical redox process using radicals coupled with nitrogen-vacancy centers in nanodiamonds. *ACS Nano* **14**, 12938–12950 (2020).
